# Biological, Behavioral and Physiological Consequences of Drug-Induced Pregnancy Termination at First-Trimester Human Equivalent in an Animal Model

**DOI:** 10.3389/fnins.2019.00544

**Published:** 2019-05-29

**Authors:** Christina Camilleri, Rebecca M. Beiter, Lisett Puentes, Paula Aracena-Sherck, Stephen Sammut

**Affiliations:** ^1^Department of Psychology, Franciscan University of Steubenville, Steubenville, OH, United States; ^2^School of Medicine, Universidad San Sebastián, Conceptión, Chile

**Keywords:** animal models, locomotor behavior, anhedonia, vaginal impedance, pregnancy, mifepristone, misoprostol, medical abortion

## Abstract

Given the significant physiological changes that take place during and resulting from pregnancy, as well as the relative absence of such information in relation to pregnancy termination, this study investigated the potential for developing a valid animal model to objectively assess the biological, physiological and behavioral consequences of drug-induced pregnancy termination. Female Long-Evans rats were divided into four groups (*n* = 19–21/group), controlling for drug [mifepristone (50 mg/kg/3 ml, i.g.)/misoprostol (0.3 mg/kg/ml, i.g.) or vehicle (1% Carboxymethylcellulose Sodium/0.2% Tween^®^ 80 suspension, i.g.)] and pregnancy. Drug administration took place on days 12–14 of gestation (days 28–40 human gestational equivalent). Vehicle was administered to the controls on the same days. Parameters measured included rat body weight, food intake, vaginal impedance, sucrose consumption/preference, locomotor activity, forced swim test, and home-cage activity. At the termination of the study, rats were deeply anesthetized using urethane, and blood, brain, and liver were collected for biochemical analysis. Following drug/vehicle administration, only the pregnancy termination group (pregnant, drug) displayed a significant decrease in body weight, food intake, locomotor activity-related behaviors and home-cage activity relative to the control group (non-pregnant, vehicle). Additionally, the pregnancy termination group was the only group that displayed a significant reduction in sucrose consumption/preference during Treatment Week relative to Pre-Treatment Week. Vaginal impedance did not significantly decrease over time in parous rats in contrast to all other groups, including the rats in the pregnancy termination group. Biochemical analysis indicated putative drug- and pregnancy-specific influences on oxidative balance. Regression analysis indicated that pregnancy termination was a predictor variable for body weight, food intake and all locomotor activity parameters measured. Moreover, pertaining to body weight and food intake, the pregnancy termination group displayed significant changes, which were not present in a group of naturally miscarrying rats following pregnancy loss. Overall, our results appear to suggest negative biological and behavioral effects following pregnancy termination, that appear to also be distinct from natural miscarriage, and potential benefits of parity pertaining to fecundity. Thus, our findings indicate the importance for further objective investigation of the physiological and behavioral consequences of medical abortion, in order to provide further insight into the potential implications in humans.

## Introduction

The potential for abortion-related consequences on mental health has been extensively debated in the scientific literature for more than 20 years, with little clear resolution (Tsoi et al., 1976; Gibbons, 1984; Adler et al., 1992; Thorp et al., 2005; Major et al., 2009; Coleman, 2011; Reardon, 2018). These consequences include an increased risk of mood disorders (including depression), anxiety (Cougle et al., 2003; Fergusson et al., 2006; Pedersen, 2008), substance abuse (Reardon et al., 2004a; Fergusson et al., 2006, 2008; Dingle et al., 2008) and suicide (Reardon et al., 2004b; Thorp et al., 2005; Fergusson et al., 2006; Luo et al., 2018). Given that approximately 20% of pregnancies in the United States end in abortion (Jones and Kooistra, 2011; Finer and Zolna, 2016), the seriousness of the potential mental health consequences, and the clinical difficulties associated with their treatment and/or prevention (Crosby et al., 2011; Lau and Rapee, 2011; Kupfer et al., 2012), it becomes necessary to objectively investigate any potential links between abortion and specific negative consequences that may arise from the procedure.

A number of recent studies have sought to investigate the comorbid relationship between abortion and psychopathological behaviors (e.g., depression, anxiety etc.) with reports of positive (Reardon and Cougle, 2002; Cougle et al., 2003; Reardon et al., 2004a; Thorp et al., 2005; Dingle et al., 2008; Fergusson et al., 2008; Pedersen, 2008; Mota et al., 2010; Coleman, 2011; Huang et al., 2012; Luo et al., 2018), negative (Foster et al., 2015; Biggs et al., 2016b) or no relationship (Rees and Sabia, 2007; American Psychological Association, 2008; Robinson et al., 2009; Warren et al., 2010; Munk-Olsen et al., 2011; Biggs et al., 2015, 2016a; Foster et al., 2015; van Ditzhuijzen et al., 2018). These conflicting findings are likely to arise due to the retrospective nature of most studies, differences in the follow-up period of the subjects involved in the study after the abortion procedure took place (Coleman, 2011; Coleman et al., 2012; Reardon, 2014; Biggs et al., 2016a) or the perspective of interpretation of those utilizing the data (Reardon, 2018). Irrespective of the reason, further investigation is warranted to address these issues.

As the process of pregnancy termination (abortion) in both the early and late stages of pregnancy involves a significant disturbance (Hamoda and Templeton, 2010; Lee et al., 2010) to the complex normal physical course of events associated with a normal physical state (pregnancy) (Hill and Pickinpaugh, 2008), it is reasonable to also expect physiological consequences, including an impact on reproduction (Lv et al., 2012). Among the normal changes that take place during pregnancy are those to the hypothalamic-pituitary-adrenal (HPA) axis (Lindsay and Nieman, 2005; Duthie and Reynolds, 2013), which is crucial for the control of various hormones, including glucocorticoids, and has been implicated in depression (Pariante and Lightman, 2008; Belvederi Murri et al., 2014; Juruena, 2014; Ancelin et al., 2017). Therefore, interrupting the normal course of changes in the HPA axis makes it plausible that pregnancy termination may result in physiological alterations leading to changes in mental health, irrespective of whether the abortion is medical or surgical. Relative to surgical abortion, previous work in rats has reported reduced protection from the influence of carcinogens in rats that have their fetuses removed via hysterectomy at mid-pregnancy (day 12) relative to those allowed to deliver and nurse their litters (Russo and Russo, 1980; Sinha et al., 1988; Yang et al., 1999; Rajkumar et al., 2001; Russo et al., 2005). This work appears to support human data reflecting a link between abortion and breast cancer development (Yuasa and MacMahon, 1970; Lanfranchi, 2005, 2014; Russo et al., 2005; Naieni et al., 2007; Xing et al., 2010; Huang et al., 2013; Lanfranchi and Fagan, 2014a,b).

In the case of medical abortion, consideration needs to be given to the pharmacological effects of mifepristone (RU486), in addition to any procedural consequences. Mifepristone is utilized to induce cervical ripening (Radestad et al., 1990), along with misoprostol or gemeprost (agonists at prostaglandin E_1_ receptor), which induce uterine contractions (Wing et al., 1995). Mifepristone produces its actions both by modulating progesterone action (as a progesterone receptor antagonist or partial agonist) and blocking the glucocorticoid receptors (GR) (Spitz and Bardin, 1993). It has also been reported to increase cytokine expression, which is thought to be a contributing factor to the mechanism of action of mifepristone in the termination of pregnancy (Li et al., 2004). Furthermore, increased levels of inflammatory cytokines have been reported under stressful conditions and may be a factor in triggering depression (Nguyen et al., 1998; Ishikawa et al., 2001; Steptoe et al., 2007; Shapira-Lichter et al., 2008; Kumar et al., 2011). Moreover, as a result of its actions on the GR, mifepristone has been reported to potentiate cytokine-induced depression-like behavior in rats, as measured by various parameters, including sweet-solution (saccharin) intake and locomotor behavior (Wang et al., 2011).

At the biochemical level, lowered levels of antioxidants (e.g., glutathione) and increased oxidative stress in the blood, peripheral tissue and the brain have been consistently reported in animal models of depression (Maes et al., 2011). An imbalance in oxidative stress has also been reported as a result of depression in humans (Liu et al., 2015). Redox alterations brought about by diverse insults are reflected by changes in the redox potential of the redox pair glutathione (GSH) and oxidized glutathione (GSSG) (Schafer and Buettner, 2001; Alzoubi et al., 2012; Schiavone et al., 2013), as increased levels of reactive oxygen species (ROS) can lead to an increase in the production of GSSG (Lushchak, 2012). An increase of lipid peroxidation end products, such as malondialdehyde (MDA), has also been observed as a sign of redox imbalance (Halliwell and Chirico, 1993). Given the potential for physiological consequences of pregnancy termination, it would be reasonable to expect alterations in the GSH/GSSG balance, toward a more oxidizing potential, as well as an increase in lipid peroxidation end products.

Collectively, the evidence appears to indicate the potential for physiological and biochemical changes arising from the termination of pregnancy, which may cause significant behavioral and psychological changes that could ultimately lead to various mental health consequences. The challenging nature of prospective studies on this subject in humans demands for a more objective methodology for measuring the physiological/biochemical and behavioral changes associated with the procedure of abortion. This would aid in providing some clarity into any potential causal links between the procedure and the development of psychological disorders. Thus, we proposed that the examination of pregnancy termination in an animal model, as a means to further our understanding of the physiological and behavioral consequences of abortion in a more controlled setting, with less unknown variables, would potentially provide clearer insight into the repercussions of the abortion procedure in humans.

Our work sought to create a valid animal model in order to examine the impact of treatments known to induce the pharmacological termination of pregnancy relative to sham treatments in rats. Given that medical abortions in humans are performed up to the first 10 weeks of pregnancy, the pregnancy termination for this study was performed at mid-term (days 12–14) of the rat’s gestational period (∼23 days). This compares to days 28–40 (4–6 weeks) in human embryonic development (Witschi, 1962; O’Rahilly, 1979; Hill, 2019). Our study measured behaviors characteristic of symptoms associated with depression, including, but not limited to, sucrose consumption/preference (as a measure of anhedonia; Papp et al., 1991; Willner, 1991), spontaneous locomotor activity (as a measure of psychomotor activity; Willner, 1991), immobility in the forced swim test (as a measure of behavioral despair; Porsolt et al., 1977; Castagné et al., 2010; Slattery and Cryan, 2012), as well as oxidative stress (as a biochemical outcome associated with behavioral alterations; Schafer and Buettner, 2001; Maes et al., 2011; Alzoubi et al., 2012; Schiavone et al., 2013; Liu et al., 2015).

## Materials and Methods

### Drugs

Mifepristone and misoprostol (neat oil) were purchased from Cayman Chemical (Ann Arbor, MI, United States). Urethane, Tween^®^ 80, 5,5′-dithiobis(2-nitrobenzoic acid), 2-thiobarbituric acid (TBA), L-glutathione reduced (GSH) and glutathione reductase from baker’s yeast were purchased from Sigma-Aldrich (St. Louis, MO, United States). Carboxymethylcellulose sodium (CMC-Na), 2-vinylpyridine, NADPH, 1-chloro-2,4-dinitrobenzene and trichloroacetic acid (TCA) were purchased from VWR (Philadelphia, PA, United States). All other chemicals were of the highest grade commercially available.

### Subjects

Female Long-Evans rats (*n* = 81) were carefully bred with male rats of the same species and raised in-house, avoiding any inbreeding. The original breeder pairs were purchased from Hilltop Lab Animals (Scottdale, PA, United States). All animal protocols were approved by the Franciscan University Institutional Animal Care and Use Committee (Protocol Number: 2013-01) and adhere to the Guide for the Care and Use of Laboratory Animals published by the USPHS. Animals were housed on Aspen shavings (Nepco^®^), were single-housed (beginning 9 weeks of age) and positioned in such a way that they could see, hear and smell other animals of the same species, under a 12/12 h light-dark cycle (Lights on: 2.15 a.m. or 3.15 a.m.) and controlled temperature and humidity (20–26**°**C, 30–70% relative humidity), with *ad libitum* access to standard laboratory chow (RMH 1800, LabDiet) and water, aside from the 1 h duration of the experiment. Animal behaviors were monitored daily as an indicator of their health and well-being (NIH, 2016).

### Experimental Procedure

Following single-housing, rats were acclimated to training tips on their water bottles in the home-cage, similar to those used during the experimental procedure itself. The rats continued with access to these tips in their home-cages until the end of the experiment. Five weeks prior to breeding, rat weight and impedance measurements were initiated. While rat weight was measured throughout the entire length of the experiment, impedance, measured using a Vaginal-Estrous Cycle-Monitor (MK-11, Stoelting, Wood Dale, IL, United States), was only collected until the rats were bred or assigned to a non-breeding control group. Food weight was measured beginning the week before breeding and continued through the end of the experiment. Sucrose consumption/preference and locomotor testing commenced 3 weeks prior to breeding (14 weeks of age). Following this acclimation period, the rats were assigned to an experimental group and bred accordingly (17 weeks of age). Drug or vehicle was administered at mid-term of gestation (Treatment Week; see below under “*Chemical termination, experimental groups and drug administration*”). A forced swim test (FST) was carried out 3 days post-partum (D3PP; Day 17 (D17) of gestation for rats undergoing pregnancy termination and their controls, or 3 days post-birth for rats giving natural birth and their controls). Home-cage activity was monitored on D11 of gestation (day prior to drug/vehicle administration), as well as D3PP. Sucrose consumption/preference and locomotor parameters continued to be measured for 12 weeks post-breeding. Impedance was also re-measured for 2 weeks (11 and 12 weeks post-breeding and equivalent days for control groups), immediately prior to tissue collection. For tissue collection, rats were deeply anesthetized using urethane (1.5–2 g/kg/ml, i.p.) (Field et al., 1993) and decapitated, and blood, liver and brain were collected for further analysis (see Supplementary Figure 1).

### Breeding

Vaginal impedance was measured daily (∼3.5 h prior to the start of the dark cycle), as described under “*Experimental procedure*” to determine estrus (Singletary et al., 2005). A peak in impedance was considered as an indication of estrus and was not present in pregnant rats (Bartos, 1977; Taradach, 1982; Singletary et al., 2005). Rats not showing consistent cycles (*n* = 13), as measured by vaginal impedance, were removed from the study prior to breeding and never underwent treatment. Female rats (17 weeks of age), whose impedance indicated estrus, were paired with a fertile male at the beginning of the dark cycle of the day of estrus (Yang et al., 2000). The male was removed after 8 h and the female was checked for signs of mating. The presence of a vaginal plug and daily weight gain were considered as presumptive evidence of pregnancy. Day 1 (D1) of pregnancy was considered to be the day following breeding.

### Chemical Termination, Experimental Groups, and Drug Administration

Methodology pertaining to pharmacological pregnancy termination, drug dosing, preparation, and administration was adapted from He et al. (2003) and Cabrol et al. (1991). Dosage for mifepristone and misoprostol was based on a combination of resources (He et al., 2003; Hu et al., 2012; Maddalena et al., 2013), as well as taking into consideration the doses utilized in human abortions (Fiala and Danielsson, 2006) and applying the formula for dose translation based on the Body Surface Area (BSA), as described by Reagan-Shaw et al. (2008) in order to calculate the rat equivalent dose.

In order to qualify as a successful pregnancy termination, it was necessary for blood to be evident vaginally and on cage bedding, and no further weight gain observed. Moreover, in order to avoid any potential ambiguity in interpretation of results, unsuccessful pregnancy termination procedures [e.g., problem with intragastric (i.g.) administration, *n* = 1] and natural pregnancy termination (miscarriage, *n* = 0) during the course of the pregnancy were considered as exclusion criteria for the study. The rats described under “*Results*,” pertaining to the miscarriage observations, were not bred as part of the original study.

Rats were assigned to one of four groups. The pregnant, drug group (D+P+) was administered mifepristone (50.0 mg/kg/3 ml, i.g.) on days 12, 13, and 14 of pregnancy, while misoprostol (0.3 mg/kg/ml, i.g.) was administered on day 14 of pregnancy, 2 h after the final administration of mifepristone (He et al., 2003). Drugs were administered in a 0.5–1 ml volume of a CMC-Na (1%) and Tween^®^ 80 (0.2%) suspension. The same mifepristone and misoprostol protocol was also administered to non-pregnant, age-matched female rats (D+P-: non-pregnant, drug group). Pregnant, vehicle (D-P+) and non-pregnant, vehicle (D-P-) control groups were administered vehicle (1% CMC-Na/0.2% Tween^®^ 80 suspension) on the same days (D12–14) as the D+P+ and D+P- groups.

### Behavioral Testing

The behavioral studies described below were performed on all rat groups.

#### Forced Swim Test (FST) and Home-Cage Activity

The forced swim test has been extensively utilized as a measure of behavioral despair and the efficacy of antidepressants (Porsolt et al., 1978a,b; Cryan et al., 2002; Slattery and Cryan, 2012). Non-depressed rats placed in a cylinder containing water display swimming and climbing activity, while reduced mobility reflects behavioral despair (Porsolt et al., 1978b). The experiment was conducted in a plexiglass cylinder (20 cm in diameter, made in-house) filled with water to a depth of 30 cm with sufficient space from the top of the cylinder to prevent the rats from escaping during the test. Water temperature was set at 23–25**°**C. Rats were placed in the water-cylinder for a 5 min test session on D3PP and swimming behavior was recorded using video cameras. The forced swim test was conducted during the light phase of the light/dark cycle (Castagné et al., 2010). Forced swim test videos were processed using Smart^®^ software (Panlab Harvard Apparatus) assessing passive behavior: immobility (when a rat remained floating in the water, making only the necessary movements to keep its head above water) (Cervo et al., 1988); and active behaviors: swimming (when rats made active swimming motions) and climbing (when rats made vigorous movements with its forepaws in and out of the water, usually directed against walls) (Detke and Lucki, 1995; Slattery and Cryan, 2012).

For home-cage activity recordings, rats were recorded using cameras, in their home-cages, for the first 5 min of the dark cycle under red LED lighting. Recordings were conducted on D11 (Pre-Treatment) and D3PP (Post-Treatment). Home-cage activity videos were also processed using Smart^®^ software, measuring periods of immobility.

#### Sucrose Consumption/Preference

As rats are more active during the dark period, both sucrose consumption/preference and locomotor tests were conducted in the first hour of the dark cycle (Laakso et al., 1995; Navarre et al., 2010), twice a week, on the same days each week.

Consumption/preference of a sweet palatable solution, such as sucrose or saccharin, has been utilized as a measure of changes in the sensitivity of the reward system, specifically, a reduction in consumption/preference being reflective of anhedonia (Katz, 1981; Papp et al., 1991; Grønli et al., 2005; Wang et al., 2009; Hayase, 2011).

The testing involved a two-bottle choice test containing plain water or 2% sucrose solution. Sucrose consumption/preference was measured for 1 h, using the Drinking/Feeding module of the PhenoMaster (TSE Systems, Chesterfield, MO, United States). Following the hour of sucrose access, the rats were returned to their home cages. Rats not consuming greater than 1.0 g of sucrose solution prior to breeding were eliminated from the study, as a lack of consumption could mask any potential treatment (drug, pregnancy) effect.

#### Locomotor Activity

Altered locomotor activity has also been utilized as a measure of depression (Willner, 1991; Grønli et al., 2005; Kumar et al., 2011; Wang et al., 2011; Zhu et al., 2011). Recordings were carried out concurrently with the sucrose consumption/preference testing and were measured using the InfraMot module of the PhenoMaster (TSE Systems, Chesterfield, MO, United States).

Both sucrose consumption/preference and locomotor activity data were processed using the TSE PhenoMaster V5.0.1 program (TSE Systems, Chesterfield, MO, United States). The parameters analyzed were comprised of **activity time/time active**, defined as the time during which the rat moved above the threshold (TSE Systems, Chesterfield, MO, United States) of 6cm/s (determined from previous tests in laboratory); **distance active**, defined as the distance moved during activity time; **overall speed**, defined as distance in activity time over total time (TSE Systems, Chesterfield, MO, United States), as well as **rearings**, defined as standing on hind legs for a duration >3 s (Horowitz et al., 1997).

Further analysis was conducted pertaining to activity in the corners of the cage. Corners were defined as 12 light beams in the x-axis by 6 light beams in the y-axis per corner. The corners were labeled as NW, NE, SW, SE, with the front of the cage (furthest from the wall) being the two east corners. Given the presence of the bottles in the NW corner and that on average, the rats spent less than 5% (relative to total testing time and total time in corners) of their testing time in this corner, the analyses only take into consideration the NE, SW, and SE corners. Time spent in each corner as a percentage of the total testing time was calculated for all groups.

### Tissue Collection

At the end of the experiment, the rats were deeply anesthetized using urethane (1.5–2 g/kg/ml, i.p.) (Field et al., 1993) and decapitated. Prior to the decapitation, blood samples were collected from the portal vein into a blood collection vial (VWR, BD367983). Blood-containing vials were then centrifuged in a clinical centrifuge (Clinaspin, VUL-6C) at 3000 rpm for 30 min and serum was aliquoted into 0.5–1 ml samples. Immediately following euthanasia, livers were dissected and perfused with NaCl (0.9% W/V) to remove all blood, and brains were dissected. Serum and tissues were frozen at -80°C until further analysis.

### Measures of Oxidative Stress

#### Preparation of Serum Samples

Serum samples were thawed on ice and aliquoted for measurement of the total glutathione pool (reduced plus oxidized, GSH+GSSG), GSSG, thiobarbituric acid substances (TBARS) and glutathione *S*-transferase (GST) activity. Aliquots were used directly for assaying GSH+GSSG or GST activity, while an aliquot was alkylated with 1M 2-vinylpyridine for at least 2 h at room temperature in the dark for the GSSG assay. TBARS assay was conducted on samples deproteinized with 0.24M TCA and incubated for 10 min at 4°C; protein precipitate was removed by centrifugation at 10,000 *g* for 10 min at 4°C.

#### Preparation of Brain and Liver Samples

For the analysis of brain and liver tissue, samples were thawed on ice, weighed, and homogenized in five volumes of 0.154M KCl by eight strokes in a Dounce Wheaton B homogenizer. GST activity was assayed directly in homogenates, while GSH+GSSG and GSSG were measured in homogenates deproteinized with 30% W/V TCA and neutralized with NaOH (5M). Removal of precipitated protein was achieved by centrifugation at 14,000 *g* for 10 min at room temperature. GSSG levels were determined in deproteinized homogenates following incubation with 1M 2-vinylpyridine for at least 2 h at room temperature in the dark. TBARS assay was conducted on samples deproteinized with 0.24M TCA and incubated for 10 min at 4°C; protein precipitate was removed by centrifugation at 10,000 *g* for 10 min at 4°C.

#### Spectrophotometric Measurements

Spectrophotometric measurements were conducted utilizing Shimadzu UV160U or GENESYS 10S Series UV-VIS (Thermo Fisher Scientific) spectrophotometers. Glutathione pool was assayed as previously described (Tietze, 1969; Griffith, 1980), using known GSH concentration solutions as standard. TBARS assay was performed as previously reported (Ohkawa et al., 1979) and reported as μmol-eq malondialdehyde (MDA) per liter, using the molar extinction coefficient for the MDA-TBA adduct (1.56 × 10^5^ M^-1^cm^-1^). GST activity was assayed continuously for 1–2 min at room temperature as previously described (Habig et al., 1974), in the presence of saturating concentrations of 1-chloro-2,4-dinitrobenzene as substrate (final 1 mM) and GSH as cofactor (final 4 mM) in 100 mM phosphate buffer (pH 6.5). Enzymatic activity was reported as nmol/min/ml of serum or nmol/min/g wet weight for liver and brain, using the molar extinction coefficient for the *S*-glutathionylated product (9.6 mM^-1^cm^-1^).

### Statistical Analysis

Analyses were conducted on all data (n_D+P+_ = 20, n_D+P-_ = 21, n_D-P+_ = 19 and n_D-P-_ = 21). One rat in the pregnant, drug group (D+P+) died 3 days post-pregnancy termination. The data collected from this rat to that point was still included in the analysis, as it did not deviate in any way from the behaviors of the rest of the group. In order to investigate whether or not the biochemical parameters differed significantly if collected closer to the time of the drug/vehicle administration and pregnancy termination/delivery relative to tissue collection, potentially warranting further investigation, each group also consisted of 1–2 rats whose blood, brain and liver samples were collected on D3 post-partum/pregnancy termination. Independent *t*-tests of the data with or without these data points indicated no significant differences. Data analysis was conducted using SigmaPlot 11.0 (Systat Software, Inc.). A Two-Way Repeated Measures (RM) ANOVA with one factor repetition (week) was utilized to determine if the groups were significantly different from each other prior to Treatment Week (sucrose/water consumption, rearings, distance active, percentage time active, and overall speed). A Two-Way RM ANOVA with one factor repetition (time: day or week) was utilized to analyze the following parameters: rat weight and food intake (daily); sucrose and water consumption (weekly average; Pre-Treatment vs. Treatment Week). Sucrose and water consumption were additionally analyzed using a One-Way ANOVA (within Treatment Week). Rearings, distance active, percentage time active, and overall speed were also analyzed using a One-Way ANOVA. A Two-Way ANOVA was utilized to analyze vaginal impedance (pre-breeding vs. post). A One-Way ANOVA was utilized to analyze immobility time in the FST (*n* = 11–12 per group) across the groups and percentage time immobile in home-cage activity (*n* = 4–7 per group). Where applicable, *post hoc* analysis was completed using a Tukey Test. A Kruskal-Wallis One-Way ANOVA on Ranks was utilized to analyze the biochemical variables, followed by Dunn’s *post hoc* analysis where appropriate. Backward Stepwise Elimination Regression was utilized to determine the relationship between various biological, behavioral and biochemical variables. Two models were utilized in our analysis: model 1 included drug and pregnancy as binomial predictor variables, while model 2 introduced abortion as an additional possible binomial predictor (i.e., drug, pregnancy and abortion). Various biochemical variables were also included as additional predictors in both models, with the exception of the home-cage activity. The contribution of oxidative (see below under “*Oxidative stress markers*”) and non-oxidative (see below under “*GST activity*”) glutathione (GSH) consumption were analyzed separately. Differences were considered significant at *p* < 0.05 for all analyses.

## Results

### Biological Parameters

#### Rat Weight

**FIGURE 1:**
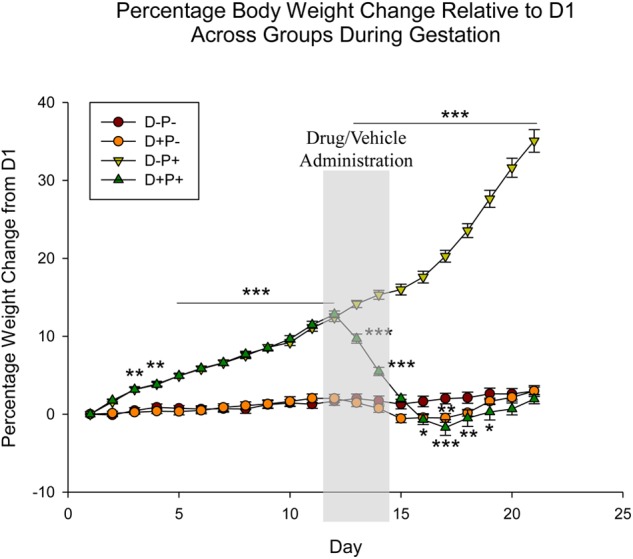
Percentage rat body weight change across gestation period relative to Day 1 (D1) of pregnancy. Following pregnancy termination, rats in the D+P+ group showed significant weight loss, decreasing below baseline (D-P-). *n* = 19–20 rats/group; ^∗^*p* < 0.05, ^∗∗^*p* < 0.01, ^∗∗∗^*p* < 0.001 (Relative to D-P-). Data is expressed as mean ± SEM. Shaded area indicates days of drug/vehicle administration.

Analysis of percentage rat weight change (relative to D1 of the gestation period) revealed a significant difference across both animal group [*F*(3, 1443) = 192.36] and gestational day [*F*(20, 1443) = 186.20], as well as the interaction between group and gestational day [*F*(60, 1443) = 193.97], all *p* < 0.001. Beginning on Day 3 (D3) of the gestation period, *post hoc* analysis indicated a significant increase (*p* < 0.01; Figure 1) in percentage weight change in the rats that carried their pregnancy to full-term (D-P+) relative to both non-pregnant groups (D-P- and D+P-). This significant increase in percentage weight continued throughout the rest of the gestation period (Relative to D-P- and D+P- on D4–21: *p* < 0.001). Rats whose pregnancy was terminated at mid-term (D+P+) experienced an initial similar significant increase in percentage weight change (relative to both D-P- and D+P- on D3–4: *p* < 0.01; D5–12: *p* < 0.001). However, this increase was only until the administration of first injection of mifepristone (D12).

Beginning on D13, rats in the D+P+ group experienced a steady and significant decrease in percentage weight relative to the D-P+ group (D13–17: *p* < 0.001), with the maximum percentage weight loss being D17. Starting on D16, the percentage weight was also significantly lower than the D-P- group (D16, 19: *p* < 0.05; D18: *p* < 0.01 D17: *p* < 0.001). The percentage weight of the D+P+ group following D19 remained below that of the D-P- group until D22. These differences, however, were not significant (*p* > 0.05). Additionally, the D+P+ group also remained significantly lower than the D-P+ group through full-term (D21, *p* < 0.001).

Non-pregnant rats who received mifepristone and misoprostol (D+P-) also experienced a decrease in percentage weight following drug administration; however, the decrease was only significantly lower than the percentage weights of the control group (D-P-) on D16 (*p* < 0.05) and D17 (*p* < 0.01). The D+P- group remained below the percentage weights of the D-P- group until D20.

##### Rat weight: comparison of full-term pregnancy (D-P+), medical abortion (D+P+) and miscarriage (spontaneous abortion)

Interestingly, the observations just described pertaining to changes in rat weight in the D+P+ group appear to be specific to rats who underwent a drug-induced termination, but not to rats that experienced a natural miscarriage (spontaneous abortion). A group of rats (*n* = 5) that was being bred for the maintenance of the colony experienced a spontaneous abortion (*n* = 4 between D13 and 16, *n* = 1 at D6 of gestation). A pregnancy was considered miscarried if the rat weight relative to D1 of gestation was equal to or less than the previous day for more than 2 days, and did not display a return to normal weight (potentially indicative of pseudopregnancy; Kennedy and Mitra, 1963; Dewar, 1964). The day of miscarriage was considered as the last day of weight gain.

Analysis of the miscarriage data relative to the D-P+ (full-term pregnancy) and D+P+ (medical abortion) groups revealed significant effects of group [*F*(2, 798) = 123.43], day [*F*(20, 798) = 120.30] and the interaction between group and day [*F*(40, 798) = 170.33], all *p* < 0.001. *Post hoc* analysis revealed no significant difference between the three groups through D12 (*p* > 0.05). As previously described, the rat weights of the D-P+ group were significantly higher than those of the D+P+ group beginning D13 through D21 (all *p* < 0.001). Following D13, the rat weights of the miscarriage group stabilized, showing no significant differences (all *p* > 0.05) between each day relative to D13, as well as to the previous day.

As a result of the stabilization in rat weight that occurred following the miscarriage, beginning D16 through D21, the miscarriage group was significantly lower than the rats that carried the pregnancy to full-term (D-P+; D16: *p* < 0.05; D17–21: *p* < 0.001).

**FIGURE 2:**
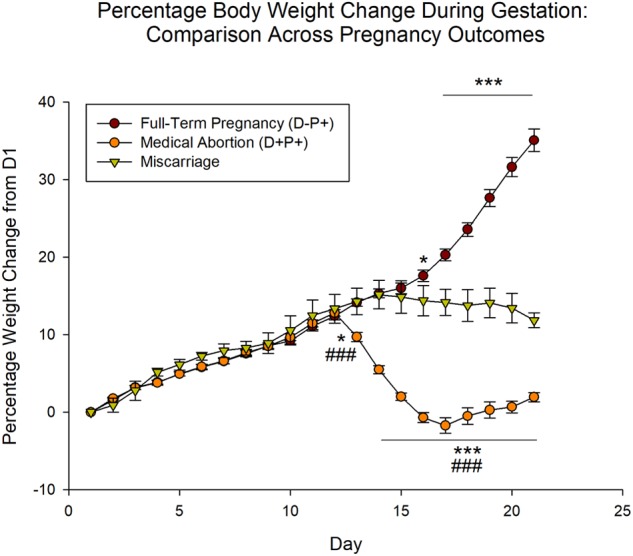
Percentage rat body weight change during gestation period relative to Day 1 (D1) across pregnancy outcomes. Unlike rats undergoing pregnancy termination, which displayed a significant weight loss, the body weight of rats that naturally miscarried did not decrease significantly, but stabilized following the miscarriage. *n* = 19–20 rats/group (D+P+ and D-P+); *n* = 5 rats (miscarriage group). ^∗^*p* < 0.05, ^∗∗∗^*p* < 0.001 (Relative to Miscarriage Group); ^###^*p* < 0.001 (Relative to D-P+). Data is expressed as mean ± SEM.

The miscarriage group also showed significant differences relative to the rats that underwent a pregnancy termination (D+P+). Specifically, the latter group displayed a significant weight loss relative to the miscarriage group starting D13 through D21 (D13: *p* < 0.05; D14–21: *p* < 0.001) (Figure 2).

#### Food Intake

**FIGURE 3:**
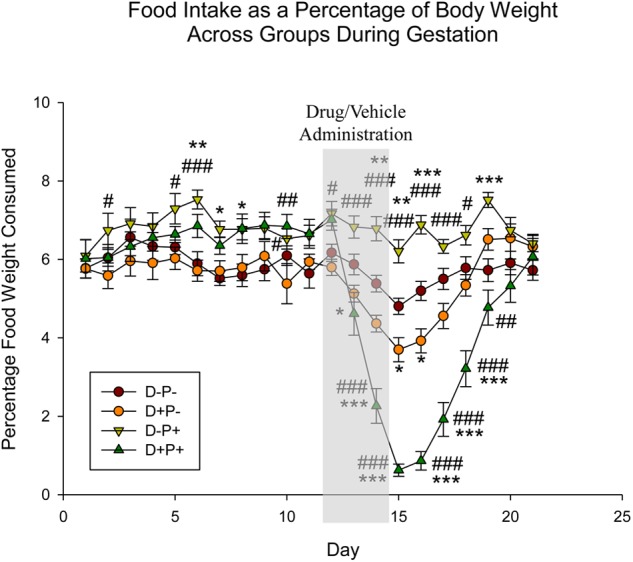
Food intake as a percentage of body weight across gestation period. Pregnancy termination induced a significant reduction in food intake. *n* = 15–17 rats/group; ^∗^*p* < 0.05, ^∗∗^*p* < 0.01, ^∗∗∗^*p* < 0.001 (Relative to D-P-); ^#^*p* < 0.05, ^##^*p* < 0.01, ^###^*p* < 0.001 (Relative to D+P-). Data is expressed as mean ± SEM. Shaded area indicates days of drug/vehicle administration.

Food intake was analyzed as a percentage of rat weight, given that rats will consume food in proportion to their weight. Statistical analysis indicated a significant difference across both animal group [*F*(3, 1180) = 42.50] and gestational day [*F*(20, 1180) = 28.33], as well as the interaction between group and day [*F*(60, 1180) = 9.18], all *p* < 0.001. Days prior to D12 revealed random significant differences (*p* < 0.05) between the two pregnant groups (D-P+ and D+P+) and the two non-pregnant groups (D+P- and D-P-). However, starting D13, food intake in the D+P+ group was significantly reduced relative to the D-P+ group until D20 (D13–19: *p* < 0.001; D20: *p* < 0.05). In the rats whose pregnancy was terminated (D+P+), there was a significant decrease in food intake relative to the control group (D-P-) following the pregnancy termination on D13 to D18 (D13: *p* < 0.05; D14–18: *p* < 0.001; Figure 3). Moreover, rats whose pregnancy was terminated (D+P+) consumed significantly less food than the non-pregnant rats who received drug (D+P-) following drug administration (D14–18: *p* < 0.001, D19: *p* < 0.01). Although food intake was also reduced in the drug group (D+P-) relative to D-P-, it was only significant on D15–16 (*p* < 0.05).

##### Food intake: comparison of full-term pregnancy (D-P+), medical abortion (D+P+) and miscarriage (spontaneous abortion)

Similar to the rat weight, the observations just described pertaining to food intake appear to be specific to rats who underwent a drug-induced termination, but not to rats that experienced a natural miscarriage. Analysis of the data comparing food intake (*n* = 4; one rat was not included in analysis due to hoarding of food, which did not enable the accurate measurement of food intake) across the various pregnancy outcomes (D-P+, D+P+ and miscarriage), setting the day of miscarriage as D_0_, and aligning this day with D12 (first day of drug/vehicle administration, also assigned as D_0_ for the purpose of this analysis) in the D+P+ and D-P+ groups, revealed a significant difference across group [*F*(2, 248) = 39.90], day [*F*(8, 248) = 20.61] and the interaction of group and day [*F*(16, 248) = 22.44], all *p* < 0.001.

**FIGURE 4:**
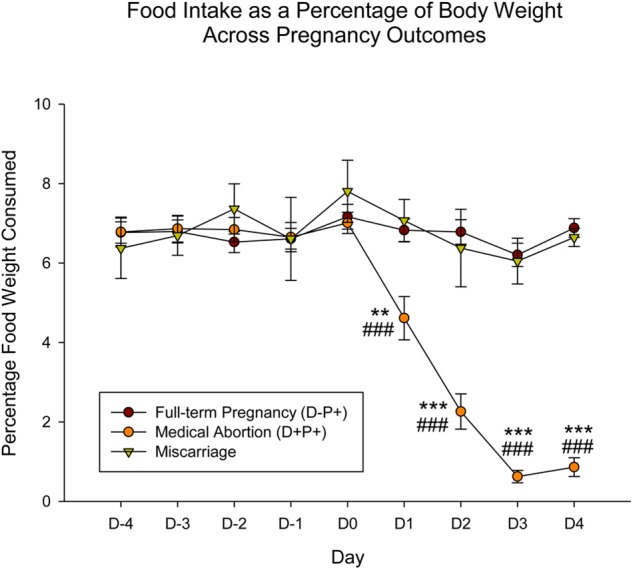
Food intake as a percentage of body weight across gestation period across pregnancy outcomes. In contrast to the significant decrease in food intake displayed by rats in the D+P+ group, rats that naturally miscarried did not display any change in food intake. Pregnancy termination induced a significant reduction in food intake. *n* = 15 rats/group (D+P+ and D-P+); *n* = 4 rats (miscarriage group). ^∗∗^*p* < 0.01, ^∗∗∗^*p* < 0.001 (Relative to Miscarriage Group); ^###^*p* < 0.001 (Relative to D-P+). Data is expressed as mean ± SEM.

*Post hoc* analysis revealed no significant difference in food intake in the miscarriage and D-P+ groups across both group and day from D_-4_ through D_4_ (all *p* > 0.05). There was also no significant difference in the D+P+ group, both across day and relative to the other two groups from D_-4_ through D_0_ (all *p* > 0.05). Beginning D_1_ through D_4_, food intake for the D+P+ group was significantly lower than both the miscarriage (D1: *p* < 0.01; D2–4: *p* < 0.001) and the D-P+ groups (all *p* < 0.001) (Figure 4).

#### Vaginal Impedance

**FIGURE 5:**
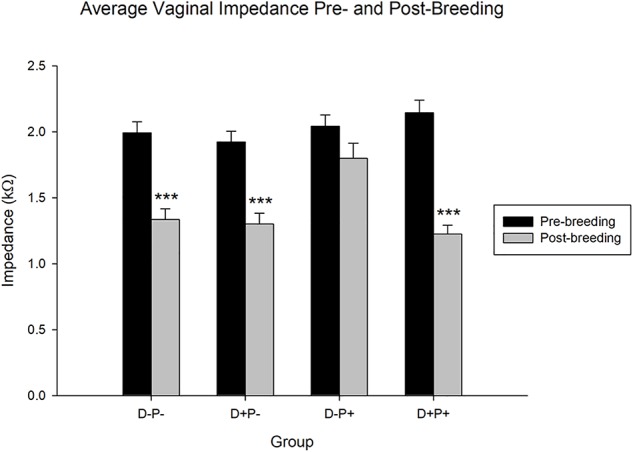
Average vaginal impedance (kΩ) pre- and post-breeding. All groups with the exception of the D-P+ group showed a significant decrease in the average vaginal impedance over time. Data points include the impedance of rats from *n* = 19–21 rats/group for pre-breeding and *n* = 11–13 rats/group for post-breeding. Data is expressed as mean ± SEM. ^∗∗∗^*p* < 0.001 (Relative to pre-breeding levels).

The collective average impedance of each group was analyzed comparing the measurements collected prior to breeding (pre-breeding) with 11 and 12 weeks post-breeding (post-breeding) and equivalent days for control groups. Statistical analysis revealed a significant difference in vaginal impedance across time [*F*(1, 2503) = 67.20, *p* < 0.001], as well as experimental group [*F*(3, 2503) = 3.70, *p* < 0.05] and the interaction between group and time [*F*(3, 2503) = 3.15, *p* < 0.05, Figure 5].

*Post hoc* analysis indicated no significant difference (*p* > 0.05) in pre-breeding impedance measurements between the groups. However, the same test revealed that the average vaginal impedance levels of all groups except the rats that carried their pregnancy to full-term (D-P+) decreased significantly post-breeding (all *p* < 0.001) relative to their pre-breeding levels. Thus, the rats that carried their pregnancy to full-term and delivered (D-P+) did not experience a significant decrease (*p* > 0.05) in their vaginal impedance over the timeframe of the experiment. Moreover, while the post-breeding vaginal impedance of the D-P-, D+P-, and D+P+ groups were not significantly different from each other (*p* > 0.05), the vaginal impedance of the D-P+ group was significantly higher post-breeding (all *p* < 0.05) relative to the other three groups (D-P-, D+P-, D+P+).

### Behavioral Parameters

#### Comparison of Behaviors Prior to Treatment Week

Analysis of the data pertaining to sucrose/water consumption, rearings, distance active, percentage time active and overall speed, in weeks prior to treatment indicated no significant difference between the experimental groups in any of these behaviors (all *p* > 0.05).

#### Sucrose and Water Consumption/Preference

**FIGURE 6:**
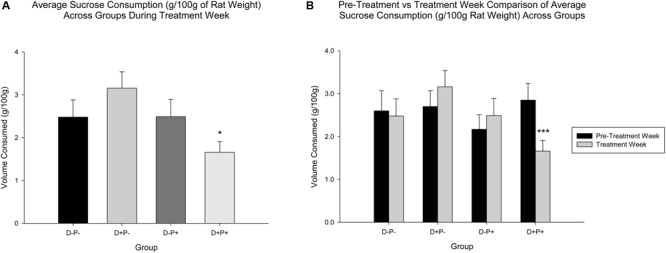
**(A)** Average sucrose consumption (g/100 g rat weight) during treatment week. Sucrose consumption was significantly reduced only in the pregnancy termination group (D+P+) relative to the D+P- group. *n* = 19–21 rats/group; ^∗^*p* < 0.05. **(B)** Average sucrose consumption (g/100 g rat weight) in pre-treatment vs. treatment week. The D+P+ group was the only group with a significant reduction in average sucrose consumption, during treatment week relative to pre-treatment week. *n* = 15–17 rats/group; ^∗∗∗^*p* < 0.001. Data is expressed as mean ± SEM.

Pertaining to sucrose consumption (g/100 g rat weight), analysis indicated a significant difference between groups [*F*(3, 77) = 2.85, *p* < 0.05] in Treatment Week. *Post hoc* analysis revealed a significant difference in sucrose consumption between D+P+ and D+P- (*p* < 0.05). Sucrose consumption for the D+P+ group in Treatment Week was not significantly different relative to any other group (*p* > 0.05) (Figure 6A).

A comparison of Treatment Week relative to Pre-Treatment Week, however, revealed no significant differences across group [*F*(3, 61) = 1.43, *p* > 0.05) or week [*F*(1, 61) = 1.13, *p* > 0.05], but a significant interaction of group and week [*F*(3, 61) = 5.17, *p* < 0.01]. *Post hoc* analysis revealed that sucrose consumption was significantly reduced in Treatment Week relative to Pre-Treatment Week, only in the D+P+ group (*p* < 0.001). All other differences across the weeks, within the other groups (D-P-, D+P-, D-P+) were not significant (all *p* > 0.05) (Figure 6B).

Water consumption was not significantly different during Treatment Week across groups [*F*(3, 77) = 0.91, *p* > 0.05]. There was also no significant difference in water consumption across group, week and the interaction between group and week when comparing Treatment Week to Pre-Treatment Week [group: *F*(3, 61) = 0.27; week: *F*(1, 61) = 1.34, group × week: *F*(3, 61) = 0.85, all *p* > 0.05].

#### Rearings

**FIGURE 7:**
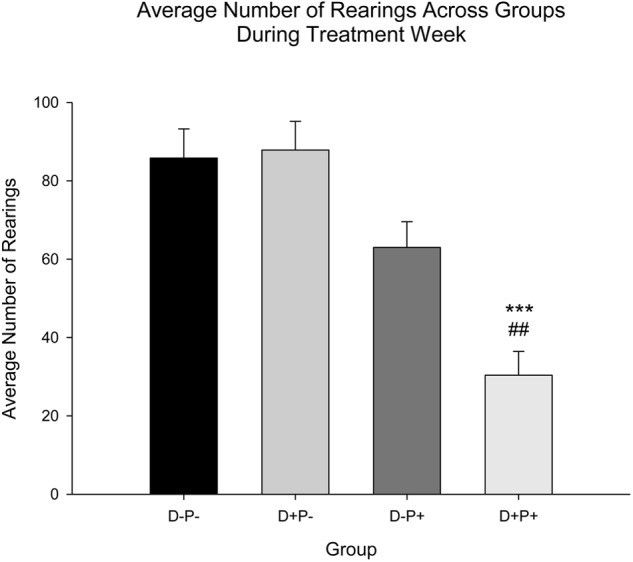
Average number of rearings during treatment week. The number of rearings was only significantly reduced during Treatment Week in the pregnancy termination group (D+P+) relative to all other groups (D-P-, D+P-, D-P+). *n* = 19–21 rats/group; ^∗∗∗^*p* < 0.001 (Relative to D-P- and D+P-); ^##^*p* < 0.01 (Relative to D-P+). Data is expressed as mean ± SEM.

Relative to the number of rearings, there was a significant difference between the treatment groups [*F*(3, 77) = 15.09, *p* < 0.001]. *Post hoc* analysis revealed that rats in the pregnancy termination group (D+P+) displayed a significant decrease in number of rearings during Treatment Week relative to all other groups (D-P- and D+P-: *p* < 0.001; D-P+: *p* < 0.01; Figure 7).

#### Distance Active, Percentage Time Active, and Overall Speed

**FIGURE 8:**
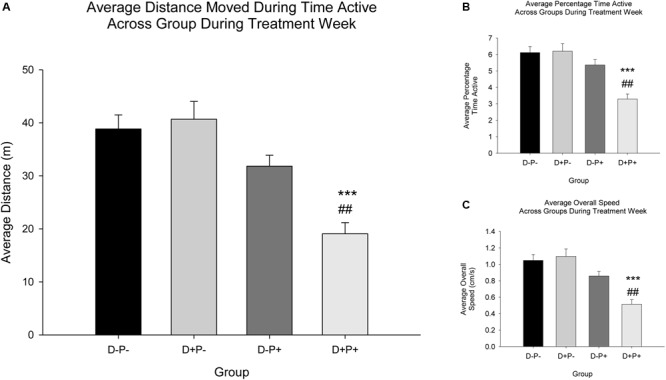
Locomotor parameters across groups during treatment week. **(A)** Average distance moved during time active (m); **(B)** Average percentage time active; **(C)** Average overall speed (cm/s). A significant decrease in all three parameters **(A–C)** was observed in the D+P+ group relative to all other groups (D-P-, D+P-, D-P+). ^∗∗∗^ *p* < 0.001 (Relative to D-P- and D+P-); ^##^*p* < 0.01 (Relative to D-P+). Data is expressed as mean ± SEM.

Additional locomotor parameters measured included distance active (Figure 8A), percentage time active (Figure 8B), and overall speed (Figure 8C). Analysis revealed a significant difference between treatment groups during Treatment Week for distance active [*F*(3, 76) = 14.15], percentage time active [*F*(3, 76) = 13.35], and overall speed [*F*(3, 76) = 14.18], all *p* < 0.001. *Post hoc* analysis revealed a significant decrease in all locomotor parameters (distance active, percentage time active and overall speed) in rats whose pregnancy was terminated (D+P+) relative to all other groups (all parameters: Relative to D+P- and D-P-*p* < 0.001; D-P+ *p* < 0.01).

#### Behaviors Post-delivery

The changes in sucrose consumption (including relative to the previous week), rearings, distance active, percentage time active and overall speed reported above, pertaining to the D+P+ group following pregnancy termination, were not observed in the D-P+ group following delivery relative to the non-pregnant control group (D-P-) (all *p* > 0.05).

#### Testing Cage Corner Activity

**FIGURE 9:**
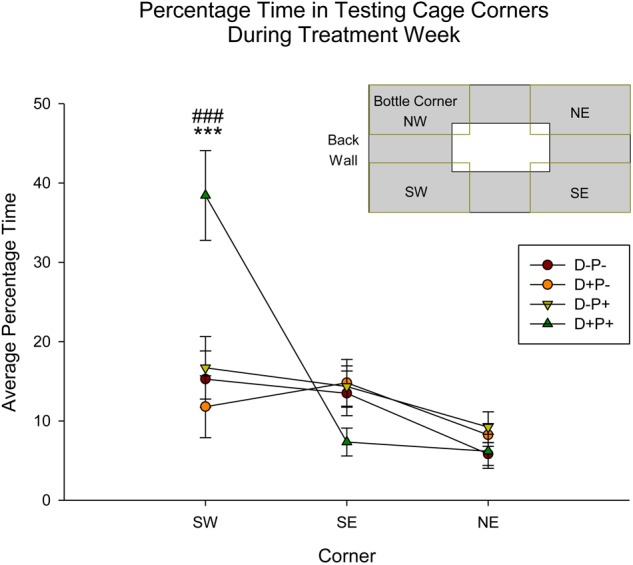
Percentage time in specific testing cage corners during treatment week. The D+P+ group spent significantly more time in the SW corner of the testing cage relative to all other groups, as well as relative to the SE and NE corners. ^∗∗∗^*p* < 0.001 (Relative to all groups); ^###^*p* < 0.001 (Relative to SE and NE corner). Data is expressed as mean ± SEM. Inset: Cage and corner orientation.

Corner activity (NE, SE, SW; see Figure 9 inset), measured as time spent in corner as a percentage of the total testing time, during Treatment Week was significantly different between groups [*F*(3, 152) = 5.29, *p* < 0.01], corner [*F*(2, 152) = 13.92, *p* < 0.001] and the interaction of group and corner [*F*(6, 152) = 6.12, *p* < 0.001]. *Post hoc* analysis revealed a significant difference between the D+P+ group and all other groups (D-P-, D-P+, D+P-) within the SW corner (all *p* < 0.001; Figure 9). Moreover, the percentage time spent in the SW corner by the D+P+ group was also significantly higher than the two other corners (both *p* < 0.001). All other comparisons were not significant (all *p* > 0.05).

#### Home-Cage Activity

**FIGURE 10:**
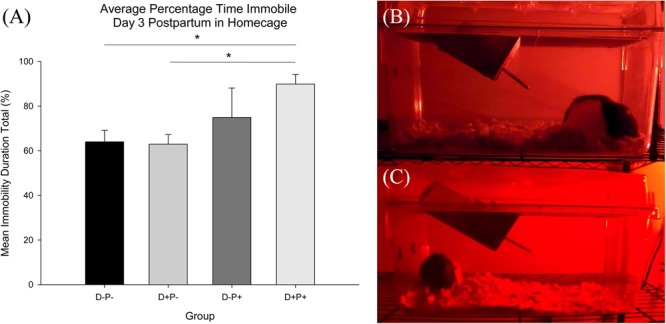
**(A)** Average percentage time immobile in home-cage. Percentage immobility time was significantly reduced in the D+P+ group relative to D-P- and D+P-. ^∗^*p* < 0.05. Data is expressed as mean ± SEM. **(B,C)** Freeze-frame shot of home-cage behavior recording during dark cycle day 3 postpartum. **(B)** D+P+; hunched posture typical of D+P+ group and **(C)** D-P+; mother nursing pups.

Analysis of the home-cage activity (percentage time immobile) revealed no significant difference between any of the groups Pre-Treatment (D11) [*F*(3, 19) = 0.39, *p* > 0.05]. However, analysis did indicate a significant difference in home-cage immobility between groups Post-Treatment [D3PP; *F*(3, 19) = 4.69, *p* < 0.05]. *Post hoc* analysis revealed a significant increase in immobility in the D+P+ group relative to both D-P- and D+P- (both *p* < 0.05, Figure 10). There was no significant difference between the D+P+ and D-P+ groups (*p* > 0.05).

#### Forced Swim Test

There was no statistically significant difference between the groups in percentage immobility duration in the Forced Swim Test on Day 3 post-partum [D-P-: M = 23.82, SEM = 4.47; D+P-: *M* = 30.67, SEM = 5.19; D-P+: *M* = 32.39; SEM = 4.90; D+P+: *M* = 31.73, SEM = 5.03; *F*(3, 43) = 0.67], *p* > 0.05. Potential confounds and limitations pertaining to this test will be explained in the *Discussion*.

### Biochemical Parameters

#### Oxidative Stress Markers

**FIGURE 11:**
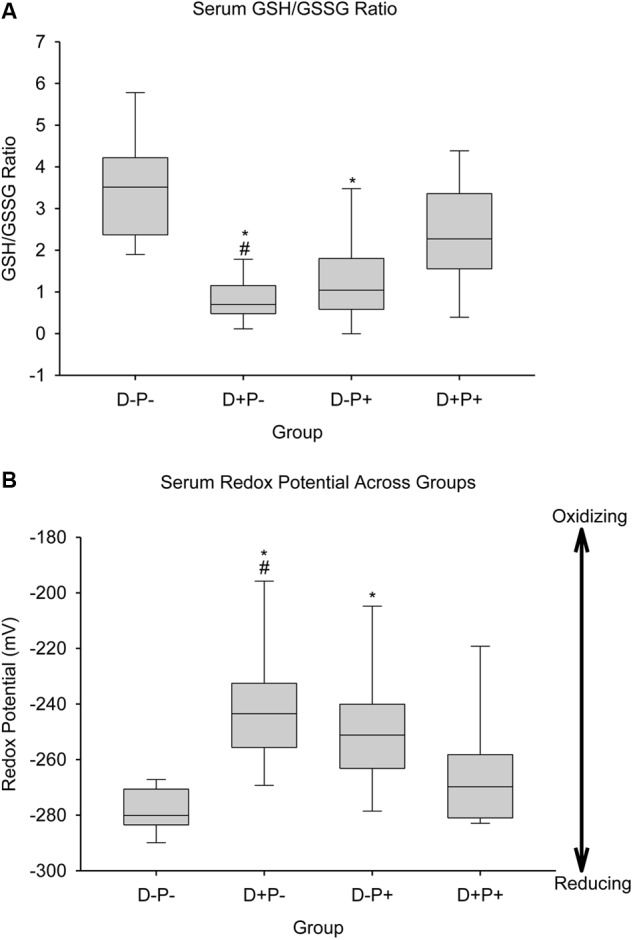
Biochemical parameters reflecting oxidative consumption of glutathione. **(A)** Serum GSH/GSSG ratio and **(B)** Serum redox potential of the glutathione pair. Data for serum GSH/GSSG ratio and redox potential of the serum glutathione pair are shown as boxes (25–75% percentiles) and whiskers (min to max values) for each group, with inside line indicating the median of each group. *n* = 10–13 rats per group; ^∗^*p* < 0.05 (Relative to D-P-); ^#^*p* < 0.05 (Relative to D+P+).

Markers of oxidative stress relative to the glutathione pool [GSH-reduced (GSH), GSH-oxidized (GSSG), GSH/GSSG ratio (Ratio), and redox potential (Redox)] and lipid peroxidation end products (TBARS) were measured in serum (s), brain (b), and liver (l) samples collected from the rats at the end of the study, which are summarized in Table 1. Analysis indicated a significant difference between groups in sGSH (*H* = 20.85; *p* < 0.001), sGSSG (*H* = 13.46; *p* < 0.01), sRatio (*H* = 25.45; *p* < 0.001), and sRedox (*H* = 24.06; *p* < 0.001). *Post hoc* analysis revealed a significant difference (*p* < 0.05) between D-P- and both D+P- and D-P+, as well as between D+P+ and D+P- in sGSH, sRatio (Figure 11A) and sRedox (Figure 11B). There was also a significant difference (*p* < 0.05) in sGSSG between D+P- and both D-P- and D+P+. All other comparisons were non-significant across the groups (*p* > 0.05).

**Table 1:** Descriptive statistics for biochemical parameters.

Variable	Non-pregnant rats				Pregnant rats			
	Vehicle (D*-*P-)		Mifepristone + Misoprostol (D+P-)		Vehicle (D-P+)		Mifepristone + Misoprostol (D+P+)	
	Mean	SEM	Mean	SEM	Mean	SEM	Mean	SEM
**SERUM**				
GSH, μM	0.9280	0.0793	0.3422^a^	0.0717	0.4017^a^	0.0795	0.7319^b^	0.1230
GSSG, μM	0.2802	0.0184	0.3970^a^	0.0197	0.3472	0.0235	0.3080^b^	0.0216
GSH to GSSG ratio	3.4749	0.3626	0.8362^a^	0.1471	1.3108^a^	0.3225	2.3638^b^	0.3834
E_GSH/GSSG_, mV	–277.6905	2.2339	–240.4316^a^	6.3688	–248.6352^a^	6.5411	–265.8473^b^	6.4425
TBARS, μM	42.1967	5.9313	36.7164	6.4920	28.8131	2.0795	31.4829	2.2471
GST, nmol/min/ml	60.1852	15.0490	58.1439	7.4877	38.3333	3.6904	56.8452	11.6680
**LIVER**				
GSH, nmol/g wet weight	240.6786	17.3101	251.7901	11.5562	259.3759	19.3164	204.3045	21.6294
GSSG, nmol/g wet weight	41.1411	4.0781	35.2433	2.8511	35.2275	2.4654	35.2654	4.4622
GSH to GSSG ratio	6.6412	0.7364	7.7493	0.6735	8.0949	0.9344	6.4569	0.8170
E_GSH/GSSG_, mV	–157.2991	2.2987	–160.7683	1.4945	–160.6804	2.6360	–154.1215	3.0619
TBARS, nmol/g wet weight	4.7877	0.4620	4.0565	0.6081	4.8678	0.5072	4.3801	0.6081
GST, nmol/min/g wet weight	84.6651	3.1142	84.2924	2.1706	88.8174	4.0749	79.7573	4.5911
**BRAIN**				
GSH, nmol/g wet weight	50.4490	3.3123	42.4967	2.0894	44.3366	3.1387	45.5379	4.2530
GSSG, nmol/g wet weight	4.1055	0.1586	3.9348	0.1168	4.2035	0.2329	4.1421	0.2016
GSH to GSSG ratio	12.2441	0.5759	10.8598	0.4934	10.6391	0.6453	10.7332	0.7808
E_GSH/GSSG_, mV	–147.7523	1.3114	–143.8969	1.1685	–143.8665	1.6515	–143.4015	3.1804
TBARS, nmol/g wet weight	1.7528	0.1349	2.0533	0.1570	1.8129	0.1010	1.9994	0.1626
GST, nmol/min/g wet weight	5.0364	0.1710	5.1232	0.1886	4.9960	0.1607	4.9828	0.1660

*Redox parameters were measured in samples of serum, liver and brain from pregnant and non-pregnant rats that underwent pharmacological treatment with either vehicle or mifepristone and misoprostol. Tissues were collected at the end of the study and measurements were performed as described in Materials and Methods. Mean and SEM values are shown from n = 13–15 rats/group (brain and liver) and n = 7–12 rats/group (serum). ^*a*^p < 0.05 (Relative to D-P-). ^*b*^p < 0.05 (Relative to D+P-).*

#### GST Activity

As a marker of non-oxidative consumption of GSH, GST activity was assayed at the end of the study in serum, brain and liver samples. Statistical analysis did not reveal any significant differences between the different treatment groups (*p* > 0.05; Table 1).

### Multiple Regression Analysis

In order to evaluate potential correlations between the biological, behavioral and biochemical parameters measured in this study and potential predictor variables for the behaviors reported, multiple linear regression analyses were conducted using the backward stepwise elimination method. Biological and behavioral parameters during Treatment Week were considered dependent variables (outcomes). Detailed effect sizes (β-values) with their *p*-values are shown in the supplemental data included with this article (see Supplementary Tables 1–15), while Tables 2, 3 summarize the significant variables associated with oxidative consumption included as final predictors of each behavioral variable.

**Table 2:** Summary of treatment variables (drug, pregnancy, abortion) as final significant predictors for biological and behavioral variables.

Variable	Dependent variable influenced	β	*p*	Effect
Drug	Rat weight (model 1)	–14.011	<0.001	Negative
	Food intake (both models)	–4.515	<0.001	
Pregnancy	Rat weight (both models)	21.947	<0.001	Positive
	Food intake (model 2)	1.653	<0.001	
	Rearings (both models)	–50.015	<0.001	Negative
	Distance active (model 1)	–13.024	0.002	
	Time active (model 1)	–1.575	0.011	
	Overall speed (model 1)	–0.351	0.002	
	Home-cage immobility (model 1)	20.964	0.003	Positive
Abortion	Rat weight (model 2)	–24.540	<0.001	Negative
	Food intake (model 2)	–4.072	<0.001	
	Rearings (model 2)	–33.658	0.009	
	Distance active (model 2)	–17.660	<0.001	
	Time active (model 2)	–2.573	<0.001	
	Overall speed (model 2)	–0.476	<0.001	
	Home-cage immobility (model 2)	23.559	0.002	Positive

*Effect size (β value) and significance of Drug, Pregnancy and Abortion on various biological and behavioral parameters, as indicated by backward stepwise elimination regression analysis. Detailed data from each model is shown in the Supplementary Tables 1–7, 15.*

**Table 3:** Summary of final significant biochemical (oxidative consumption) predictors for biological and behavioral variables.

	Variable	Dependent variable influenced	β	*p*	Effect
Serum	GSH	Rat weight (model 1)	–7.218	0.004	Negative
		Food intake (model 1)	–1.239	0.035	
	GSSG	Rat weight (model 2)	–19.579	0.001	Negative
		Food intake (model 1)	7.154	0.007	Positive
	TBARS	Rearings (both models)	–0.650	0.011	Negative
Brain	E_GSH/GSSG_	Rat weight (model 1)	0.370	0.044	Positive
	TBARS	Rearings (model 1)	–16.621	0.046	Negative
Liver	No variable was found to influence the dependent variables measured				

*Effect size (*β-*value) of biochemical variables related to oxidative consumption of GSH in serum, brain and liver on various biological and behavioral parameters, as indicated by backward stepwise elimination regression analysis. Detailed data from each model is shown in the Supplementary Tables 1–7.*

The analysis revealed various relationships between drug, pregnancy and abortion and the dependent variables (biological and behavioral) previously mentioned (i.e., rat weight, impedance, food intake, rearings, distance active, percentage time active and overall speed). Our results from the analysis of variables involved in oxidative consumption of GSH (Table 2) indicated that drug was a predictor of rat weight only in model 1 (excludes abortion as a predictor variable). However, pregnancy was found to be a predictor of rat weight regardless of whether or not abortion was considered as a predictor (model 1: *R*^2^= 0.760, model 2: *R*^2^= 0.923). Food intake was predicted by drug in both models (model 1: *R*^2^= 0.826; model 2: *R*^2^= 0.896), while it was predicted by pregnancy in model 2 only (when abortion was included as a predictor variable). Similar relationships were present with the behavioral variables (i.e., rearings, distance active, percentage time active and overall speed). Pregnancy predicted distance active, percentage time active and overall speed only when abortion was not included as a variable in the model (i.e., model 1; *R*^2^ range = 0.147–0.214). However, pregnancy predicted rearings in both models (model 1: *R*^2^= 0.473, model 2: *R*^2^= 0.510). The inclusion of abortion as a predictor variable (i.e., model 2, *R*^2^ range = 0.264–0.923) indicated that this variable predicted rat weight, food intake and all behavioral variables measured.

Pertaining to the biochemical predictor variables, rat weight was found to be predicted by serum GSH and brain redox potential in model 1, while it was only predicted by serum GSSG levels when abortion was included as a possible predictor (model 2) (Table 3). Moreover, food intake was predicted by both serum GSH and GSSG levels when abortion was not included in the model (model 1) (Table 3). The biochemical variables measured were not significant predictors of the distance active, percentage time active and overall speed. However, serum TBARS predicted rearings in both models, while brain TBARS was a predictor only in model 1.

Regression analysis with the non-oxidative biochemical variables (see Supplementary Tables 8–14) revealed identical effects of similar magnitude to that of the analysis with the oxidative variables (Table 2). The only exceptions to this were impedance that was found to be predicted by abortion (in model 2) and rearings that was predicted by pregnancy only in model 1. Serum, liver and brain GST were not found to influence any of the dependent variables measured.

Regression analysis with home-cage immobility as the dependent variable indicated that while pregnancy was a significant predictor in model 1 (abortion not included as a predictor), when abortion was added as a potential predictor (model 2), pregnancy no longer predicted the home-cage immobility behavior. In this model (model 2), abortion was the only predictor of the observed behavior (see Supplementary Table 15). Biochemical parameters were not measured in these rats, and therefore, were not included in the regression analysis.

Thus, the results from the regression analyses appear to corroborate the observations from the previously mentioned analyses.

## Discussion

This study investigated the biological, behavioral and physiological consequences of pharmacologically terminating a pregnancy at mid-term (first-trimester human equivalent) in an animal model. Taken together, our analyses appear to indicate a significant effect of pregnancy termination on the biological (rat weight, food intake, vaginal impedance), physiological (oxidative balance) and most especially, behavioral parameters (sucrose consumption, rearings, distance active, percentage time active, overall speed) measured.

### Body Weight and Food Intake

Body weight and food intake were most notably affected in the pregnancy termination group (D+P+) relative to all other groups. Although there was a decrease in these parameters in the drug group (D+P-), the behavior in this specific group is clearly not indicative of a lack of well-being of the rat, in contrast to the D+P+ group. This is evident in the difference of the magnitude of the observed effects, as well as observations relative to grooming, exploratory behavior and general activity, which will be further discussed below under “*Home-cage behavior and locomotor activity.*” Furthermore, the specific influence of the drug-induced termination on food intake was further confirmed by the absence of similar observations in rats who naturally miscarried.

Previous literature indicates an effect of stress on both body weight and food intake. In humans, stress has been shown to be associated with both an increase and decrease in body weight and is influenced by various variables unique to the specific situations (Korkeila et al., 1998; Kivimaki et al., 2006; Stinson et al., 2018). In animals, however, stress has been primarily associated with weight loss (Alario et al., 1987; Harris et al., 2002; Dallman et al., 2003). Our findings appear to suggest a similar impact of pregnancy termination on body weight. Regarding food intake, the literature reports a variety of effects on food consumption and appetite following periods of stress or depression at both the level of the animal (Marti et al., 1994; Brown and Grunberg, 1996; Torres and Nowson, 2007) and human (Cartwright et al., 2003; Torres and Nowson, 2007; Mikolajczyk et al., 2009; Konttinen et al., 2010). Previous literature (Torres and Nowson, 2007; Harris, 2015) also indicates that in animal models, the specific effect of stress on food intake is dependent on the level of the stressor. Mild stressors have been shown to generally not alter food consumption in rats (Bertiere et al., 1984; Marti et al., 1994; Willner, 1997), whereas moderate and severe stressors have resulted in decreased food consumption (Alario et al., 1987; Monteiro et al., 1989; Krebs et al., 1996; Valles et al., 2000; Armario et al., 2004). Thus, our food intake results from the pregnancy termination group (D+P+) suggest that their behavior demonstrates a similarity to the findings observed in the case of moderate to severe stressors. Our results further indicate that the observed biological changes are distinguished from what was observed following natural miscarriages.

### Sucrose Consumption

Relative to sucrose consumption, previous studies have reported a decrease in sweet solution consumption relative to baseline and control, following various chronic mild stress protocols, which is interpreted to be indicative of a putative anhedonic effect (Willner et al., 1987; Muscat and Willner, 1992; Willner, 1997, 2017; Grippo et al., 2003, 2006; Grønli et al., 2005; Rossetti et al., 2016). Our results appear to suggest a similar effect (decrease) of pregnancy termination (D+P+) on sucrose consumption, with the effects being reported during the exposure to the stressor (Willner et al., 1987; Muscat and Willner, 1992; Grippo et al., 2003; Rossetti et al., 2016). Moreover, the findings of Grippo and colleagues (Grippo et al., 2003) indicate a recovery of the behavior following the discontinuation of the chronic mild stress protocol.

### Home-Cage Behavior, Locomotor Activity, and Corner Activity

Subjective observations of the rats in their home cages, made by the investigators, indicated the following distinct negative behaviors (NIH, 2016) in the D+P+ group following pregnancy termination, relative to all other groups: a clear reduction in grooming, an unkempt coat, a reduction in exploratory behavior (e.g., rearing, sniffing) when investigators entered the housing room, a reduction in general activity, increased immobility and the assumption of a stooped/hunched posture in the corner of the cage (see Figure 10B,C). Importantly, these parameters are indicators of a lack of a general well-being and health of the rat (NIH, 2016). Home-cage recordings were conducted in an effort to objectively capture and analyze aspects of these behaviors, most specifically, the increased immobility. The objective analysis of the recordings confirmed the subjective observations, indicating decreased activity in the home cage in the D+P+ group 3 days post pregnancy termination.

These observations also appear to be supported by the behavioral measures collected in the testing cage during the experimental testing period. This included a significant reduction in all locomotor activity parameters measured (i.e., distance active, percentage time active and overall speed, as well as rearings) the week of the pregnancy termination in the D+P+ group, relative to all other groups. Similar to body weight and food intake, previous literature indicates a variety of effects on locomotor activity following periods of stress or depression in both animals (Hooks et al., 1991; Gorka et al., 1996; Willner, 1997; Grønli et al., 2005; You et al., 2011) and humans (American Psychiatric Association, 2013). Additionally, the corner activity appears to further support the other locomotor measures, indicating a potentially increased level of anxiety-like behavior in the D+P+ group, as displayed by the significantly higher percentage of time spent in the back corner of the testing cage. This is potentially reflective of behaviors similar to those reported in open-field studies (Sestakova et al., 2013; Crumeyrolle-Arias et al., 2014; Sarkaki et al., 2015).

### FST Immobility

Relative to the forced swim test, no significant differences were observed. Given the negative behaviors reported above, as well as the effects of stress on this behavior, previously reported in the literature (Porsolt et al., 1977; Castagné et al., 2010; Slattery and Cryan, 2012), this was somewhat unexpected. However, the literature appears to be divided relative to the effects of various stressors on the forced swim test, with reports of depressant, antidepressant and no effect (Bogdanova et al., 2013), including in chronic mild stress (Harro et al., 2001; Haidkind et al., 2003; Bourke and Neigh, 2011). Therefore, it is potentially possible that the forced swim test is not sensitive to the apparent stress observed following the induced pregnancy termination procedure.

### Vaginal Impedance and Fecundity

Another parameter measured in our study was vaginal impedance, as an indicator of estrus and therefore of an increased sexual receptivity (Bartos, 1977; Taradach, 1982; Rezac, 2008; Hockey et al., 2010). The relationship between (a) the estrous cycle, (b) estrus and the associated increased sexual receptivity, (c) fecundity, and (d) the use of vaginal impedance measurements as an objective method for the purpose of fertility management has been previously documented in the literature (Singletary et al., 2005; Rezac, 2008; Luno et al., 2013).

The reduced impedance, between the beginning and the end of the experimental period, in all groups except the rats that carried the pregnancy to full-term (D-P+), appears to indicate a potential effect of pregnancy on the duration of fecundity (the ability to reproduce). Our results appear to suggest that parity is more important than gravidity for fecundity, given that the impedance of the rats whose pregnancy was terminated (D+P+) decreased significantly over the experimental time-period, yet was equivalent to the non-pregnant, vehicle group (D-P-). This potentially corroborates previous research, suggesting that the act of conceiving is not sufficient to lead to the protective effects of pregnancy in humans (Baird and Dunson, 2003; Russo et al., 2005; Lanfranchi and Fagan, 2014a) and in rats (Russo and Russo, 1980; Sinha et al., 1988; Walker et al., 2001; Russo et al., 2005). Our impedance results also appear to be in agreement with previous reports indicating that prior pregnancy reduced the infertility and impaired fecundity levels in humans (Chandra et al., 2013) and that medical abortion affected reproductive capacity and reduced the success of subsequent pregnancies in mice (Lv et al., 2012).

### Pregnancy and Oxidative Stress

Our results indicate an increased oxidative stress in the rats who carried the pregnancy to full-term (D-P+). This corresponded to a lower GSH/GSSG ratio and a higher redox potential, indicative of a lower antioxidant capacity (Schafer and Buettner, 2001; Wu et al., 2004; Maes et al., 2011; Zitka et al., 2012; Liu et al., 2015). The literature indicates that oxidative stress is associated with a negative effect on fertility (Ruder et al., 2009; Agarwal et al., 2012). However, this does not represent the full picture of oxidative stress. In our results, this is evident in the effects observed in the vaginal impedance measurements in the D-P+ group, as described above. Moreover, this appears to be consistent with research that indicates that an increase in oxidative stress is beneficial and necessary for a successful pregnancy to occur and be maintained (Jenkins et al., 2000; Wiener-Megnazi et al., 2004; Patil et al., 2007; Michel and Bonnet, 2014; Pereira et al., 2015; Mannaerts et al., 2018). While similar increases have been reported in cases of miscarriage and other pathophysiological complications of pregnancy (Al-Gubory et al., 2010; Burton and Jauniaux, 2011; Ramiro-Cortijo et al., 2016), the difference appears to be in cellular adaptations that take place in the peripheral blood, which offer protection from oxidative damage in the successful pregnancies (Jenkins et al., 2000). This could potentially reflect our biochemical observations in the serum several weeks following the pregnancy in the D-P+ group.

### Drug Administration and Oxidative Stress

Our results also indicate an increased oxidative stress in the drug group (D+P-). As previously mentioned, this corresponded to a lower GSH/GSSG ratio and a higher redox potential, indicative of a lower antioxidant capacity (Schafer and Buettner, 2001; Wu et al., 2004; Maes et al., 2011; Zitka et al., 2012; Liu et al., 2015). Although these results are similar to those of the parous rats in the experiment (D-P+), it would appear that the mechanism and reason behind such changes, and thus the overall effect, are potentially different. This appears to be supported by the observations in the vaginal impedance, which showed a decrease over time in all groups except for the D-P+ group.

From a pharmacological perspective, mifepristone acts as both a progesterone and a GR antagonist (Spitz and Bardin, 1993). A resulting effect of the latter mechanism is a disinhibition of cortisol release (Bertagna et al., 1984, 1986; Laue et al., 1990; Young, 2004). Additionally, glucocorticoids have been reported to reduce glutathione peroxidase activity (McIntosh et al., 1998; Patel et al., 2002; Beytut et al., 2018). Thus, as an antagonist of glucocorticoid receptors, mifepristone may potentially have contributed to the changes we observed in oxidative balance toward a lower antioxidant capacity in the non-pregnant, drug group (D+P-). In addition, liver drug biotransformation can lead to oxidative stress (McGill et al., 2015; Du et al., 2016); however, there were no significant changes in liver redox parameters between treatment groups, including glutathione *S*-transferase activity (a class II biotransformation enzyme), suggesting that drug biotransformation is not likely a contributor to the observed changes in redox parameters.

### Pregnancy Termination and Oxidative Stress

Given the necessity for a certain level of oxidative stress in order to maintain a pregnancy, as well as the relationship between carrying a pregnancy to full-term and fecundity (as indicated by the vaginal impedance measurements), it would appear, from our results, that the rats whose pregnancy was terminated (D+P+) are precluded from the potential benefits of pregnancy. This was potentially reflected in some of the biochemical observations in our study.

The changes in oxidative balance observed in the rats that carried their pregnancy to term (D-P+), as well as those present in the non-pregnant, drug group (D+P-) were not observed following the termination of the pregnancy (D+P+). We argue that such changes, described above, in the D-P+ group, may be indicative of potentially positive effects of carrying the pregnancy to term, while in the D+P- group, the pharmacological imbalance associated with the interaction between mifepristone and glucocorticoids may potentially be the cause of the oxidative changes observed. Thus, the absence of similarity to either pregnancy alone or drug administration alone at the biochemical level, as well as the negative behaviors observed in the pregnancy termination group (D+P+) indicate a more complex dynamic that requires more in-depth investigation of the consequences arising from and specific to the termination. Moreover, the presence of distant physiological changes relative to the stressor is supported by research showing a prolonged effect on other physiological parameters (e.g., cardiovascular change), following chronic mild stress, despite the recovery of the behavioral parameters measured (sucrose intake and activity levels) (Grippo et al., 2003).

### Limitations

As with all animal models, while the findings from our study cannot be directly extrapolated to the level of the human person, they provide the possibility of objectively and ethically investigating the putative consequences of pregnancy termination at the biological, behavioral and physiological levels.

Given the novelty of this investigation, certain limitations should be taken into consideration in the interpretation of certain results, as well as for future experiments, including the absence of physiological, histological and anatomical measures related to the reproductive system (e.g., uterus, ovary, vagina) and its health. Additionally, a further limitation to consider is the inevitable lack of precise alignment of the experimental days during which the behavioral measurements (e.g., sucrose/water consumption/preference and locomotor activity) were conducted, relative to the pregnancy termination. It is possible that the absence of significance in sucrose consumption, between the D+P+ group and both the D-P- and D-P+ groups within Treatment Week may reflect a sensitivity to the necessity for more exact alignment of this particular parameter, especially given our observations relative to Pre-Treatment Week. An additional factor for consideration, and related to timing, is that due to the nature of this experiment, there is a limited capacity for adjustment.

Another potential distinction may also arise from the fact that in other studies, rats were deprived of water (and in some cases food) for extended periods of time prior to the sucrose test (Willner et al., 1992; Wang et al., 2009). This was not an option in our study, as it would have added a significant confounding variable given the potential effects of inappropriate nutrition on pregnancy.

### Future Direction

Future experiments will focus on investigating the potential reversal of the behaviors observed in our study, including through the administration of antidepressants. Moreover, given the role of the hypothalamic-pituitary-adrenal axis and its significance in stress, the changes involved in pregnancy, glucocorticoid regulation, the perception of reward, the electrophysiological and electrochemical dynamics within the various brain regions potentially affected will also be investigated, as well as behaviors that could potentially be influenced by such altered dynamics, such as drug-addiction and maternal care of future litters. Additionally, similar experiments will be carried out to investigate the parameters addressed in this study following late-term surgical pregnancy termination.

## Conclusion

To our knowledge, our study is the first report addressing the potential biological, behavioral and biochemical effects associated with pregnancy termination in an animal model. Additionally, the findings of this study also appear to provide additional support to the current literature pertaining to the benefits of carrying a pregnancy to full-term. Moreover, we believe that our findings support the use of this model as an objective method for the investigation of potential physical (biological and physiological) and behavioral effects of induced pregnancy termination. Our findings strongly suggest that pregnancy termination at mid-term (first-trimester human equivalent) induces significant negative biological and behavioral changes in the rat. Additionally, such a procedure appears to be associated with a potential absence of beneficial effects of carrying a pregnancy to full-term. Moreover, our findings also appear to indicate a significant difference between induced pregnancy termination (medical abortion) and natural miscarriage. Our study, therefore, indicates the importance and necessity for further objective research into the abortion procedure, including at the physiological and neurophysiological levels. Such work may further our understanding and potentially shed some clarity into the potential biobehavioral impact of such a procedure at the level of the human person.

## Data Availability

Datasets are available on request. The raw data supporting the conclusions of this manuscript will be made available by the authors, without undue reservation, to any qualified researcher.

## Ethics Statement

This study was carried out in accordance with the recommendations of the Guide for the Care and Use of Laboratory Animals published by the USPHS. The protocol was approved by the Franciscan University Institutional Animal Care and Use Committee (IACUC; Protocol Number. 2013-01).

## Author Contributions

SS contributed to the conception, design, supervision and acquisition of funding of the study. SS, CC, and RB conducted the behavioral experiments. PA-S contributed to the design and supervision of the biochemistry aspect of the study. SS, CC, and PA-S performed the statistical analyses. SS and CC wrote the first draft of the manuscript. SS, CC, PA-S, and LP contributed to research investigation and writing of the biochemistry section of the manuscript. All authors contributed to manuscript revision, read and approved the submitted version.

## Conflict of Interest Statement

The authors declare that the research was conducted in the absence of any commercial or financial relationships that could be construed as a potential conflict of interest.
