# Supplementary material for: Biological, Behavioral and Physiological Consequences of Drug-Induced Pregnancy Termination at First-Trimester Human Equivalent in an Animal Model
Source: Front Neurosci. 2019 May 29;13:544. doi: 10.3389/fnins.2019.00544 (PMC6549702; doi:10.3389/fnins.2019.00544)
Supplement: Supplementary file 1 [file Table_1.DOCX]

**Supplementary Table 1. Influence of treatment (drug, pregnancy, abortion) and oxidative consumption variables on rat weight.** Effect sizes (β values) were obtained through backward stepwise regression analyses, as detailed in *Materials and methods*. Table shows the β value of each variable at the step in which it was eliminated from the model and the overall R^2^ for each model. Significant β values of variables included in the final model are shown in boldface letters and summarized in Tables 2 and 3 of the main manuscript.

| **Variable** | | **MODEL 1** | | | **MODEL 2** | | |
| --- | --- | --- | --- | --- | --- | --- | --- |
|  |  | **β** | ***p*** | **Backward step of elimination** | **β** | ***p*** | **Backward step of elimination** |
| Drug | | **-14.011** | **< 0.001** | **Not eliminated** | 1.189 | 0.532 | 5 |
| Pregnancy | | **9.166** | **< 0.001** | **Not eliminated** | **21.947** | **< 0.001** | **Not eliminated** |
| Abortion (only model 2) | |  | | | **-24.540** | **< 0.001** | **Not eliminated** |
| Serum | GSH | **-7.218** | **0.004** | **Not eliminated** | -0.859 | 0.803 | 1 |
|  | GSSG | 13.125 | 0.240 | 7 | **-19.579** | **0.001** | **Not eliminated** |
|  | E_redox_ | -0.103 | 0.301 | 6 | -0.023 | 0.351 | 7 |
|  | TBARS | 0.001 | 0.983 | 2 | 0.012 | 0.683 | 4 |
| Liver | GSH | 0.016 | 0.221 | 10 | 0.012 | 0.138 | 9 |
|  | GSSG | -0.021 | 0.770 | 3 | -0.035 | 0.772 | 2 |
|  | E_redox_ | -0.012 | 0.985 | 1 | 0.016 | 0.880 | 3 |
|  | TBARS | 0.159 | 0.725 | 4 | 0.310 | 0.180 | 8 |
| Brain | GSH | 0.608 | 0.165 | 8 | 0.253 | 0.253 | 10 |
|  | GSSG | -0.952 | 0.533 | 9 | -1.503 | 0.061 | 12 |
|  | E_redox_ | **0.370** | **0.044** | **Not eliminated** | 0.056 | 0.562 | 11 |
|  | TBARS | -1.199 | 0.490 | 5 | 0.704 | 0.465 | 6 |
| R^2^ for model | | 0.760 | | | 0.923 | | |
