# Supplementary material for: Biological, Behavioral and Physiological Consequences of Drug-Induced Pregnancy Termination at First-Trimester Human Equivalent in an Animal Model
Source: Front Neurosci. 2019 May 29;13:544. doi: 10.3389/fnins.2019.00544 (PMC6549702; doi:10.3389/fnins.2019.00544)
Supplement: Supplementary file 2 [file Table_2.DOCX]

**Supplementary Table 2. Influence of treatment (drug, pregnancy, abortion) and oxidative consumption variables on food intake.** Effect sizes (β values) were obtained through backward stepwise regression analyses, as detailed in *Materials and methods*. Table shows the β value of each variable at the step in which it was eliminated from the model and the overall R^2^ for each model. Significant β values of variables included in the final model are shown in boldface letters and summarized in Tables 2 and 3 of the main manuscript.

| **Variable** | | **MODEL 1** | | | **MODEL 2** | | |
| --- | --- | --- | --- | --- | --- | --- | --- |
|  |  | **β** | ***p*** | **Backward step of elimination** | **β** | ***p*** | **Backward step of elimination** |
| Drug | | **-4.515** | **< 0.001** | **Not eliminated** | **-2.277** | **< 0.001** | **Not eliminated** |
| Pregnancy | | -0.686 | 0.104 | 11 | **1.653** | **< 0.001** | **Not eliminated** |
| Abortion (only model 2) | |  | | | **-4.072** | **< 0.001** | **Not eliminated** |
| Serum | GSH | **-1.239** | **0.035** | **Not eliminated** | 0.312 | 0.873 | 1 |
|  | GSSG | **7.154** | **0.007** | **Not eliminated** | -2.936 | 0.338 | 9 |
|  | E_redox_ | -0.017 | 0.405 | 6 | 0.001 | 0.924 | 2 |
|  | TBARS | -0.001 | 0.953 | 1 | -0.008 | 0.369 | 7 |
| Liver | GSH | 0.007 | 0.217 | 7 | -0.0004 | 0.874 | 5 |
|  | GSSG | -0.007 | 0.889 | 2 | 0.003 | 0.820 | 6 |
|  | E_redox_ | 0.018 | 0.400 | 8 | -0.083 | 0.482 | 4 |
|  | TBARS | 0.080 | 0.527 | 3 | -0.047 | 0.694 | 3 |
| Brain | GSH | 0.070 | 0.163 | 9 | 0.005 | 0.783 | 12 |
|  | GSSG | -0.675 | 0.364 | 4 | -0.844 | 0.138 | 10 |
|  | E_redox_ | 0.045 | 0.304 | 10 | 0.079 | 0.346 | 11 |
|  | TBARS | -0.515 | 0.267 | 5 | 0.415 | 0.223 | 8 |
| R^2^ for model | | 0.826 | | | 0.896 | | |
