# Supplementary material for: Biological, Behavioral and Physiological Consequences of Drug-Induced Pregnancy Termination at First-Trimester Human Equivalent in an Animal Model
Source: Front Neurosci. 2019 May 29;13:544. doi: 10.3389/fnins.2019.00544 (PMC6549702; doi:10.3389/fnins.2019.00544)
Supplement: Supplementary file 3 [file Table_3.DOCX]

**Supplementary Table 3. Influence of treatment (drug, pregnancy, abortion) and oxidative consumption variables on sucrose consumption.** Effect sizes (β values) were obtained through backward stepwise regression analyses, as detailed in *Materials and methods*. Table shows the β value of each variable at the step in which it was eliminated from the model and the overall R^2^ for each model.

| **Variable** | | **MODEL 1** | | | **MODEL 2** | | |
| --- | --- | --- | --- | --- | --- | --- | --- |
|  |  | **β** | ***p*** | **Backward step of elimination** | **β** | ***p*** | **Backward step of elimination** |
| Drug | | -0.556 | 0.300 | 12 | 0.686 | 0.525 | 5 |
| Pregnancy | | -0.168 | 0.778 | 4 | 0.477 | 0.496 | 6 |
| Abortion (only model 2) | |  | | | -0.939 | 0.133 | 14 |
| Serum | GSH | 0.538 | 0.742 | 5 | 0.917 | 0.565 | 7 |
|  | GSSG | -1.035 | 0.795 | 3 | -2.686 | 0.429 | 8 |
|  | E_redox_ | 0.014 | 0.195 | 13 | 0.010 | 0.360 | 10 |
|  | TBARS | 0.028 | 0.054 | Not eliminated | 0.028 | 0.054 | Not eliminated |
| Liver | GSH | -0.007 | 0.314 | 10 | -0.002 | 0.578 | 12 |
|  | GSSG | 0.037 | 0.555 | 7 | 0.029 | 0.676 | 3 |
|  | E_redox_ | -0.013 | 0.676 | 11 | -0.065 | 0.232 | 11 |
|  | TBARS | 0.091 | 0.495 | 8 | 0.111 | 0.387 | 9 |
| Brain | GSH | -0.028 | 0.271 | 9 | -0.023 | 0.326 | 13 |
|  | GSSG | -0.121 | 0.846 | 2 | -0.003 | 0.997 | 1 |
|  | E_redox_ | -0.017 | 0.950 | 1 | -0.077 | 0.650 | 4 |
|  | TBARS | -0.295 | 0.585 | 6 | -0.037 | 0.954 | 2 |
| R^2^ for model | | 0.085 | | | 0.085 | | |
