# Supplementary material for: Biological, Behavioral and Physiological Consequences of Drug-Induced Pregnancy Termination at First-Trimester Human Equivalent in an Animal Model
Source: Front Neurosci. 2019 May 29;13:544. doi: 10.3389/fnins.2019.00544 (PMC6549702; doi:10.3389/fnins.2019.00544)
Supplement: Supplementary file 4 [file Table_4.DOCX]

**Supplementary Table 4. Influence of treatment (drug, pregnancy, abortion) and oxidative consumption variables on** **rearings.** Effect sizes (β values) were obtained through backward stepwise regression analyses, as detailed in *Materials and methods*. Table shows the β value of each variable at the step in which it was eliminated from the model and the overall R^2^ for each model. Significant β values of variables included in the final model are shown in boldface letters and summarized in Tables 2 and 3 of the main manuscript.

| **Variable** | | **MODEL 1** | | | **MODEL 2** | | |
| --- | --- | --- | --- | --- | --- | --- | --- |
|  |  | **β** | ***p*** | **Backward step of elimination** | **β** | ***p*** | **Backward step of elimination** |
| Drug | | -9.144 | 0.309 | 7 | 12.984 | 0.240 | 8 |
| Pregnancy | | **-50.015** | **< 0.001** | **Not eliminated** | **-31.975** | **0.003** | **Not eliminated** |
| Abortion (only model 2) | |  | | | **-33.658** | **0.009** | **Not eliminated** |
| Serum | GSH | -18.621 | 0.089 | 11 | -6.874 | 0.632 | 6 |
|  | GSSG | 53.474 | 0.328 | 6 | 21.092 | 0.743 | 4 |
|  | E_redox_ | -0.085 | 0.854 | 2 | -0.049 | 0.915 | 2 |
|  | TBARS | **-0.650** | **0.011** | **Not eliminated** | **-0.541** | **0.025** | **Not eliminated** |
| Liver | GSH | 0.052 | 0.684 | 4 | 0.051 | 0.679 | 5 |
|  | GSSG | -0.018 | 0.987 | 1 | -0.026 | 0.982 | 1 |
|  | E_redox_ | 0.299 | 0.570 | 5 | 0.373 | 0.460 | 7 |
|  | TBARS | -0.471 | 0.842 | 3 | -0.350 | 0.881 | 3 |
| Brain | GSH | 0.524 | 0.283 | 9 | 0.516 | 0.271 | 10 |
|  | GSSG | -5.519 | 0.457 | 10 | -6.831 | 0.336 | 11 |
|  | E_redox_ | 5.314 | 0.142 | 8 | 4.578 | 0.198 | 9 |
|  | TBARS | **-16.621** | **0.046** | **Not eliminated** | -11.779 | 0.147 | 12 |
| R^2^ for model | | 0.473 | | | 0.510 | | |
