# Supplementary material for: Biological, Behavioral and Physiological Consequences of Drug-Induced Pregnancy Termination at First-Trimester Human Equivalent in an Animal Model
Source: Front Neurosci. 2019 May 29;13:544. doi: 10.3389/fnins.2019.00544 (PMC6549702; doi:10.3389/fnins.2019.00544)
Supplement: Supplementary file 6 [file Table_6.DOCX]

**Supplementary Table 6.** **Influence of oxidative consumption variables on percentage time active.** Effect sizes (β values) were obtained through backward stepwise regression analyses, as detailed in *Materials and methods*. Table shows the β value of each variable at the step in which it was eliminated from the model and the overall R^2^ for each model. Significant β values of variables included in the final model are shown in boldface letters and summarized in Tables 2 and 3 of the main manuscript.

| **Variable** | | **MODEL 1** | | | **MODEL 2** | | |
| --- | --- | --- | --- | --- | --- | --- | --- |
|  |  | **β** | ***p*** | **Backward step of elimination** | **β** | ***p*** | **Backward step of elimination** |
| Drug | | -0.954 | 0.105 | 13 | 0.274 | 0.783 | 7 |
| Pregnancy | | **-1.575** | **0.011** | **Not eliminated** | -0.636 | 0.364 | 11 |
| Abortion (only model 2) | |  | | | **-2.573** | **< 0.001** | **Not eliminated** |
| Serum | GSH | -0.440 | 0.580 | 10 | -0.841 | 0.645 | 9 |
|  | GSSG | 2.281 | 0.560 | 8 | 0.143 | 0.978 | 2 |
|  | E_redox_ | -0.024 | 0.461 | 9 | -0.014 | 0.277 | 12 |
|  | TBARS | -0.029 | 0.078 | 12 | -0.020 | 0.185 | 14 |
| Liver | GSH | -0.001 | 0.850 | 4 | -0.002 | 0.584 | 10 |
|  | GSSG | -0.003 | 0.975 | 1 | -0.001 | 0.989 | 1 |
|  | E_redox_ | -0.022 | 0.757 | 3 | -0.016 | 0.816 | 3 |
|  | TBARS | 0.028 | 0.869 | 2 | 0.040 | 0.806 | 4 |
| Brain | GSH | 0.092 | 0.530 | 5 | 0.007 | 0.843 | 6 |
|  | GSSG | -0.695 | 0.161 | 11 | -0.636 | 0.183 | 13 |
|  | E_redox_ | 0.010 | 0.878 | 6 | 0.083 | 0.763 | 5 |
|  | TBARS | -0.392 | 0.515 | 7 | -0.183 | 0.742 | 8 |
| R^2^ for model | | 0.147 | | | 0.264 | | |
