# Supplementary material for: Biological, Behavioral and Physiological Consequences of Drug-Induced Pregnancy Termination at First-Trimester Human Equivalent in an Animal Model
Source: Front Neurosci. 2019 May 29;13:544. doi: 10.3389/fnins.2019.00544 (PMC6549702; doi:10.3389/fnins.2019.00544)
Supplement: Supplementary file 7 [file Table_7.DOCX]

**Supplementary Table 7.** **Influence of treatment (drug, pregnancy, abortion) and oxidative consumption variables on overall speed.** Effect sizes (β values) were obtained through backward stepwise regression analyses, as detailed in *Materials and methods*. Table shows the β value of each variable at the step in which it was eliminated from the model and the overall R^2^ for each model. Significant β values of variables included in the final model are shown in boldface letters and summarized in Tables 2 and 3 of the main manuscript.

| **Variable** | | **MODEL 1** | | | **MODEL 2** | | |
| --- | --- | --- | --- | --- | --- | --- | --- |
|  |  | **β** | ***p*** | **Backward step of elimination** | **β** | ***p*** | **Backward step of elimination** |
| Drug | | -0.162 | 0.115 | 12 | 0.045 | 0.842 | 3 |
| Pregnancy | | **-0.351** | **0.002** | **Not eliminated** | -0.189 | 0.131 | 14 |
| Abortion (only model 2) | |  | | | **-0.476** | **< 0.001** | **Not eliminated** |
| Serum | GSH | -0.107 | 0.451 | 11 | -0.284 | 0.384 | 10 |
|  | GSSG | 0.589 | 0.423 | 7 | 0.313 | 0.685 | 6 |
|  | E_redox_ | -0.006 | 0.281 | 10 | -0.002 | 0.379 | 11 |
|  | TBARS | -0.005 | 0.104 | 13 | -0.005 | 0.113 | 13 |
| Liver | GSH | -0.0003 | 0.735 | 4 | -0.001 | 0.509 | 9 |
|  | GSSG | 0.001 | 0.889 | 2 | 0.001 | 0.842 | 2 |
|  | E_redox_ | -0.001 | 0.988 | 1 | -0.0004 | 0.993 | 1 |
|  | TBARS | 0.007 | 0.829 | 3 | 0.007 | 0.822 | 4 |
| Brain | GSH | 0.002 | 0.693 | 6 | 0.003 | 0.647 | 7 |
|  | GSSG | -0.083 | 0.363 | 9 | -0.080 | 0.359 | 12 |
|  | E_redox_ | 0.036 | 0.449 | 5 | 0.022 | 0.667 | 5 |
|  | TBARS | -0.069 | 0.500 | 8 | -0.056 | 0.576 | 8 |
| R^2^ for model | | 0.214 | | | 0.265 | | |
