# Supplementary material for: Biological, Behavioral and Physiological Consequences of Drug-Induced Pregnancy Termination at First-Trimester Human Equivalent in an Animal Model
Source: Front Neurosci. 2019 May 29;13:544. doi: 10.3389/fnins.2019.00544 (PMC6549702; doi:10.3389/fnins.2019.00544)
Supplement: Supplementary file 8 [file Table_8.DOCX]

**Supplementary Table 8.** **Influence of non-oxidative consumption variables on rat weight.** Effect sizes (β values) were obtained through backward stepwise regression analyses, as detailed in *Materials and methods*. Table shows the β value of each variable at the step in which it was eliminated from the model and the overall R^2^ for each model. β values of variables included in the final model are shown in boldface letters.

| **Variable** | | **MODEL 1** | | | **MODEL 2** | | |
| --- | --- | --- | --- | --- | --- | --- | --- |
|  |  | **β** | ***p*** | **Backward step of elimination** | **β** | ***p*** | **Backward step of elimination** |
| Drug | | **-10.962** | **< 0.001** | **Not eliminated** | -2.190 | 0.121 | 4 |
| Pregnancy | | **11.516** | **< 0.001** | **Not eliminated** | **21.887** | **< 0.001** | **Not eliminated** |
| Abortion (only model 2) | |  | | | **-21.508** | **< 0.001** | **Not eliminated** |
| GST activity | Serum | -0.044 | 0.196 | 3 | -0.013 | 0.486 | 2 |
|  | Liver | 0.046 | 0.541 | 2 | 0.022 | 0.593 | 1 |
|  | Brain | 0.631 | 0.692 | 1 | 0.927 | 0.259 | 3 |
| R^2^ for model | | 0.698 | | | 0.913 | | |
