# Supplementary material for: Biological, Behavioral and Physiological Consequences of Drug-Induced Pregnancy Termination at First-Trimester Human Equivalent in an Animal Model
Source: Front Neurosci. 2019 May 29;13:544. doi: 10.3389/fnins.2019.00544 (PMC6549702; doi:10.3389/fnins.2019.00544)
Supplement: Supplementary file 9 [file Table_9.DOCX]

**Supplementary Table 9.** **Influence of non-oxidative consumption variables on food intake.** Effect sizes (β values) were obtained through backward stepwise regression analyses, as detailed in *Materials and methods*. Table shows the β value of each variable at the step in which it was eliminated from the model and the overall R^2^ for each model. β values of variables included in the final model are shown in boldface letters.

| **Variable** | | **MODEL 1** | | | **MODEL 2** | | |
| --- | --- | --- | --- | --- | --- | --- | --- |
|  |  | **Β** | ***p*** | **Backward step of elimination** | **β** | ***p*** | **Backward step of elimination** |
| Drug | | **-4.222** | **< 0.001** | **Not eliminated** | **-2.564** | **< 0.001** | **Not eliminated** |
| Pregnancy | | -0.572 | 0.234 | 3 | **1.357** | **0.004** | **Not eliminated** |
| Abortion (only model 2) | |  | | | **-3.808** | **< 0.001** | **Not eliminated** |
| GST activity | Serum | -0.010 | 0.191 | 4 | -0.004 | 0.430 | 3 |
|  | Liver | -0.006 | 0.759 | 2 | -0.007 | 0.572 | 2 |
|  | Brain | -0.069 | 0.868 | 1 | -0.018 | 0.949 | 1 |
| R^2^ for model | | 0.737 | | | 0.891 | | |
