# Supplementary material for: Biological, Behavioral and Physiological Consequences of Drug-Induced Pregnancy Termination at First-Trimester Human Equivalent in an Animal Model
Source: Front Neurosci. 2019 May 29;13:544. doi: 10.3389/fnins.2019.00544 (PMC6549702; doi:10.3389/fnins.2019.00544)
Supplement: Supplementary file 10 [file Table_10.DOCX]

**Supplementary Table 10. Influence of non-oxidative consumption variables on rearings.** Effect sizes (β values) were obtained through backward stepwise regression analyses, as detailed in *Materials and methods*. Table shows the β value of each variable at the step in which it was eliminated from the model and the overall R^2^ for each model. β values of variables included in the final model are shown in boldface letters.

| **Variable** | | **MODEL 1** | | | **MODEL 2** | | |
| --- | --- | --- | --- | --- | --- | --- | --- |
|  |  | **β** | ***p*** | **Backward step of elimination** | **β** | ***p*** | **Backward step of elimination** |
| Drug | | -13.491 | 0.159 | 4 | 17.806 | 0.066 | 5 |
| Pregnancy | | **-36.038** | **< 0.001** | **Not eliminated** | -7.852 | 0.507 | 3 |
| Abortion (only model 2) | |  | | | **-55.807** | **< 0.001** | **Not eliminated** |
| GST activity | Serum | 0.101 | 0.563 | 1 | 0.195 | 0.164 | 4 |
|  | Liver | 0.215 | 0.549 | 2 | 0.209 | 0.514 | 2 |
|  | Brain | -5.679 | 0.456 | 3 | -3.805 | 0.576 | 1 |
| R^2^ for model | | 0.294 | | | 0.435 | | |
