# Supplementary material for: Biological, Behavioral and Physiological Consequences of Drug-Induced Pregnancy Termination at First-Trimester Human Equivalent in an Animal Model
Source: Front Neurosci. 2019 May 29;13:544. doi: 10.3389/fnins.2019.00544 (PMC6549702; doi:10.3389/fnins.2019.00544)
Supplement: Supplementary file 12 [file Table_12.DOCX]

**Supplementary Table 12.** **Influence of non-oxidative consumption variables on percentage time active.** Effect sizes (β values) were obtained through backward stepwise regression analyses, as detailed in *Materials and methods*. Table shows the β value of each variable at the step in which it was eliminated from the model and the overall R^2^ for each model. β values of variables included in the final model are shown in boldface letters.

| **Variable** | | **MODEL 1** | | | **MODEL 2** | | |
| --- | --- | --- | --- | --- | --- | --- | --- |
|  |  | **β** | ***p*** | **Backward step of elimination** | **β** | ***p*** | **Backward step of elimination** |
| Drug | | -1.195 | 0.062 | 4 | -0.109 | 0.895 | 1 |
| Pregnancy | | **-1.532** | **0.022** | **Not eliminated** | -0.649 | 0.348 | 4 |
| Abortion (only model 2) | |  | | | **-2.842** | **< 0.001** | **Not eliminated** |
| GST activity | Serum | 0.003 | 0.777 | 1 | 0.006 | 0.549 | 2 |
|  | Liver | 0.021 | 0.364 | 2 | 0.016 | 0.473 | 3 |
|  | Brain | -0.643 | 0.197 | 3 | -0.543 | 0.238 | 5 |
| R^2^ for model | | 0.142 | | | 0.301 | | |
