# Supplementary material for: Biological, Behavioral and Physiological Consequences of Drug-Induced Pregnancy Termination at First-Trimester Human Equivalent in an Animal Model
Source: Front Neurosci. 2019 May 29;13:544. doi: 10.3389/fnins.2019.00544 (PMC6549702; doi:10.3389/fnins.2019.00544)
Supplement: Supplementary file 13 [file Table_13.DOCX]

**Supplementary Table 13. Influence of non-oxidative consumption variables on overall speed.** Effect sizes (β values) were obtained through backward stepwise regression analyses, as detailed in *Materials and methods*. Table shows the β value of each variable at the step in which it was eliminated from the model and the overall R^2^ for each model. β values of variables included in the final model are shown in boldface letters.

| **Variable** | | **MODEL 1** | | | **MODEL 2** | | |
| --- | --- | --- | --- | --- | --- | --- | --- |
|  |  | **β** | ***p*** | **Backward step of elimination** | **β** | ***p*** | **Backward step of elimination** |
| Drug | | -0.199 | 0.071 | 4 | -0.033 | 0.821 | 1 |
| Pregnancy | | **-0.336** | **0.004** | **Not eliminated** | -0.172 | 0.158 | 5 |
| Abortion (only model 2) | |  | | | **-0.514** | **< 0.001** | **Not eliminated** |
| GST activity | Serum | 0.001 | 0.570 | 1 | 0.001 | 0.485 | 3 |
|  | Liver | 0.003 | 0.440 | 2 | 0.003 | 0.442 | 2 |
|  | Brain | -0.093 | 0.279 | 3 | -0.090 | 0.276 | 4 |
| R^2^ for model | | 0.212 | | | 0.306 | | |
