# Supplementary material for: Biological, Behavioral and Physiological Consequences of Drug-Induced Pregnancy Termination at First-Trimester Human Equivalent in an Animal Model
Source: Front Neurosci. 2019 May 29;13:544. doi: 10.3389/fnins.2019.00544 (PMC6549702; doi:10.3389/fnins.2019.00544)
Supplement: Supplementary file 14 [file Table_14.DOCX]

**Supplementary Table 14.** **Influence of non-oxidative consumption variables on change in impedance over time.** Effect sizes (β values) were obtained through backward stepwise regression analyses, as detailed in *Materials and methods*. For this variable, model 1 was not statistically significant. Table shows the β value of each variable at the step in which it was eliminated from the model and the overall R^2^ for the model. β values of variables included in the final model are shown in boldface letters.

| **Variable** | | **MODEL 2** | | |
| --- | --- | --- | --- | --- |
|  |  | **β** | ***p*** | **Backward step of elimination** |
| Drug | | 0.252 | 0.278 | 4 |
| Pregnancy | | -0.055 | 0.845 | 3 |
| Abortion (only model 2) | | **-0.758** | **0.003** | **Not eliminated** |
| GST activity | Serum | 0.0005 | 0.886 | 2 |
|  | Liver | 0.001 | 0.882 | 1 |
|  | Brain | -0.137 | 0.384 | 5 |
| R^2^ for model | | 0.271 | | |
