# Supplementary material for: Biological, Behavioral and Physiological Consequences of Drug-Induced Pregnancy Termination at First-Trimester Human Equivalent in an Animal Model
Source: Front Neurosci. 2019 May 29;13:544. doi: 10.3389/fnins.2019.00544 (PMC6549702; doi:10.3389/fnins.2019.00544)
Supplement: Supplementary file 15 [file Table_15.DOCX]

**Supplementary Table 15.** **Influence of treatment variables (drug, pregnancy, abortion) on percentage time immobile in home-cage.** Effect sizes (β values) were obtained through backward stepwise regression analyses, as detailed in *Materials and methods*. Table shows the β value of each variable at the step in which it was eliminated from the model and the overall R^2^ for each model. β values of variables included in the final model are shown in boldface letters.

| **Variable** | **MODEL 1** | | | **MODEL 2** | | |
| --- | --- | --- | --- | --- | --- | --- |
|  | **β** | ***p*** | **Backward step of elimination** | **β** | ***p*** | **Backward step of elimination** |
| Drug | 6.308 | 0.336 | 1 | -1.058 | 0.903 | 1 |
| Pregnancy | **20.964** | **0.003** | **Not eliminated** | 11.426 | 0.187 | 2 |
| Abortion (only model 2) |  | | | **23.559** | **0.002** | **Not eliminated** |
| R^2^ for model | 0.346 | | | 0.371 | | |
